# The complex geography of domestication of the African rice *Oryza glaberrima*

**DOI:** 10.1371/journal.pgen.1007414

**Published:** 2019-03-07

**Authors:** Jae Young Choi, Maricris Zaidem, Rafal Gutaker, Katherine Dorph, Rakesh Kumar Singh, Michael D. Purugganan

**Affiliations:** 1 Center for Genomics and Systems Biology, Department of Biology, New York University, New York, NY United States of America; 2 Rice Breeding Platform, International Rice Research Institute, Los Baños, Laguna, Philippines; 3 Center for Genomics and Systems Biology, NYU Abu Dhabi Research Institute, New York University Abu Dhabi, Saadiyat Island, Abu Dhabi, United Arab Emirates; University of California Davis, UNITED STATES

## Abstract

While the domestication history of Asian rice has been extensively studied, details of the evolution of African rice remain elusive. The inner Niger delta has been suggested as the center of origin but molecular data to support this hypothesis is lacking. Here, we present a comprehensive analysis of the evolutionary and domestication history of African rice. By analyzing whole genome re-sequencing data from 282 individuals of domesticated African rice *Oryza glaberrima* and its progenitor *O*. *barthii*, we hypothesize a non-centric (i.e. multiregional) domestication origin for African rice. Our analyses showed genetic structure within *O*. *glaberrima* that has a geographical association. Furthermore, we have evidence that the previously hypothesized *O*. *barthii* progenitor populations in West Africa have evolutionary signatures similar to domesticated rice and carried causal domestication mutations, suggesting those progenitors were either mislabeled or may actually represent feral wild-domesticated hybrids. Phylogeographic analysis of genes involved in the core domestication process suggests that the origins of causal domestication mutations could be traced to wild progenitors in multiple different locations in West and Central Africa. In addition, measurements of panicle threshability, a key early domestication trait for seed shattering, were consistent with the gene phylogeographic results. We suggest seed non-shattering was selected from multiple genotypes, possibly arising from different geographical regions. Based on our evidence, *O*. *glaberrima* was not domesticated from a single centric location but was a result of diffuse process where multiple regions contributed key alleles for different domestication traits.

## Author summary

For many crops it is not clear how they were domesticated from their wild progenitors. Transition from a wild to domesticated state required a series of genetic changes, and studying the evolutionary origin of these domestication-causing mutations are key to understanding the domestication origins of a crop. Moreover, population comparisons provide insight into the relationship between wild and cultivated populations and the evolutionary history of domestication. In this study, we investigated the domestication history of *Oryza glaberrima*, a rice species that was domesticated in West Africa independent from the Asian rice species *O*. *sativa*. Using genome-wide data from a large sample of domesticated and wild African rice samples we did not find evidence that supported the established domestication model for *O*. *glaberrima*—a single domestication origin. Rather, our evidence suggests the domestication process for African rice was initiated in multiple regions of West Africa, caused potentially by the local environments and cultivation preference of people. Hence domestication of African rice was a multi-regional process.

## Introduction

Domestication of crop species represents a key co-evolutionary transition, in which wild plant species were cultivated by humans and eventually gave rise to new species whose propagation were dependent on human action [1–3]. The evolutionary origin(s) of various crop species have been the subject of considerable interest. Studying it has broadened our understanding of the early dynamics associated with crop species origins and divergence, the nature of human/plant interactions, and the genetic basis of domestication. Moreover, an understanding of the evolutionary history of crop species aids genetic mapping approaches, as well as informs plant breeding strategies.

Within the genus *Oryza*, crop domestication has occurred at least twice—once in Asia and separately in Africa. In Asia, the wild rice *O*. *rufipogon* was domesticated into the Asian rice *O*. *sativa* approximately 9,000 years ago [4]. In West Africa, the wild rice *O*. *barthii* was independently domesticated into the African rice *O*. *glaberrima* about 3,000 years ago [4]. Recent archaeological studies have also suggested that a third independent domestication event occurred in South America during pre-Columbian times, but this crop species is no longer cultivated [5].

**Fig 1:**
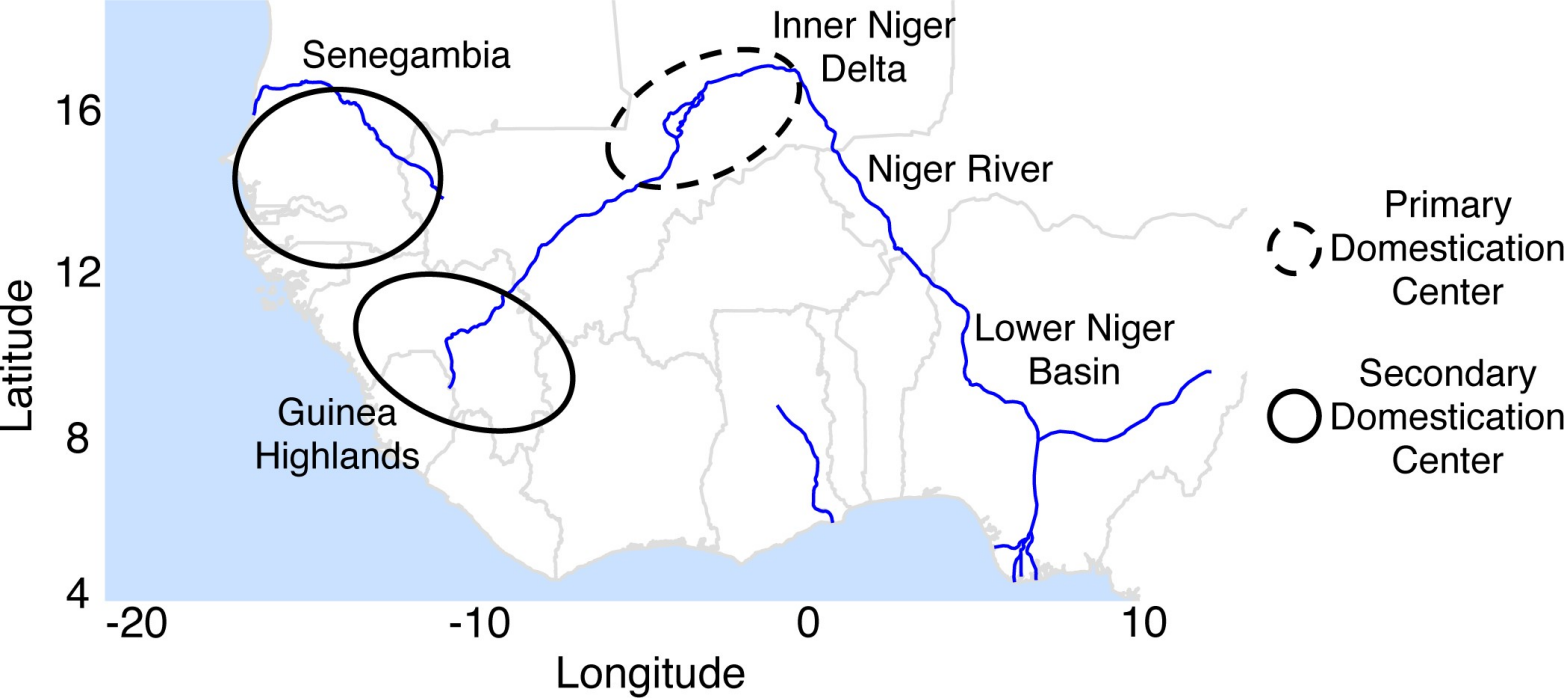
Geography of west Africa and approximate geographic regions involved in the *O*. *glaberrima* domestication model postulated by Portères [8,9].

The domestication history of Asian rice has been extensively studied both from the standpoint of archaeology [6] and genetics [7]. In contrast, much less is known about the domestication of *O*. *glaberrima*. Based on the morphology of rice grown in West Africa, the ethnobotanist Portères was the first to postulate an *O*. *glaberrima* domestication scenario [8,9], in which the inner Niger delta region in Mali as the center of domestication (Fig 1). He based this hypothesis on *O*. *glaberrima* in this area predominantly having wild rice-like traits (termed “genetically dominant characteristics” by Portères), observing loosely attached spikelets, reddish brown pericarps, and anthocyanic pigmentation. In contrast, *O*. *glaberrima* with domesticated rice-like traits (termed “genetically recessive characteristics” by Portères) were found in two geographically separated regions: (i) the Senegambia region bordering the river Sine to the north and river Casamance to the south, and (ii) the mountainous region of Guinea. Portères hypothesized the derived traits observed in *O*. *glaberrima* from Senegambia and Guinea were due to those regions being secondary centers of diversification, but the inner Niger delta region remained as the primary center of diversity for African rice. Initial archaeological excavations found ceramic impressions of rice grains in north-east Nigeria dating ~3,000 years ago, but first evidence of documented *O*. *glaberrima* has been found in the inland Niger delta at Jenne-Jeno, Mail dating ~2,000 years ago [10]. A more recent find at an excavation at the lower Niger basin north of Benin found *O*. *glaberrima* dating to ~1,600 to ~1,100 years ago, suggesting domesticated African rice had spread down the Niger river by this time from the inland Niger delta region [11].

Few population genetic studies have attempted to understand the evolutionary history and geographic structure of *O*. *glaberrima*. Microsatellite-based analysis showed genetic structure within *O*. *glaberrima* [12], suggesting the phenotypic differences observed by Portères may have stemmed from this population structure. With high-throughput sequencing technology, population genomic analysis indicated *O*. *barthii*, the wild progenitor of *O*. *glaberrima*, had evidence of population structure as well, dividing it into 5 major genetic groups (designated as OB-I to OB-V) [13]. The OB-V group from West Africa was most closely related to *O*. *glaberrima*, which caused previous researchers to suggest that this *O*. *barthii* group from West Africa was likely to be the direct progenitor of African rice [13]. Genome-wide polymorphism data also indicated that *O*. *glaberrima* had a population bottleneck spanning a period of >10,000 years, indicating a protracted period of pre-domestication related management during its domestication [14]. A recent study based on simulations detected population expansion after the bottlenecking event, to originate from the inland Niger delta suggesting the origin of *O*. *glaberrima* to be in this region [15].

While previous genome-wide variation studies have given valuable insights into the evolutionary history of *O*. *glaberrima*, they have not necessarily examined how *O*. *glaberrima* was domesticated from *O*. *barthii*. This is because the domestication history of a crop is best examined from the pattern of variation observed in genes underlying key domestication phenotypes [2]. Crop domestication accompanies a suite of traits, often called the domestication syndrome [16], which modified the wild progenitor into a domesticated plant dependent on humans for survival and dispersal [1]. In rice, these traits include the loss of seed shattering [17,18], plant architecture change for erect growth [19,20], closed panicle [21], reduction of awn length [22,23], seed hull and pericarp color changes [24,25], change in seed dormancy [26], and change in flowering time [27]. During the domestication process, it is likely that these traits were not selected at the same time and selection would have occurred in subsequent stages. Traits such as loss of seed shattering and plant erect growth would have been among the initial phenotypes humans have selected to distinguish domesticates from their wild progenitors. On the other hand, traits that improved taste and appearance of the crop, or adaptation to the local environment would likely have been favored in later diversification/improvement stages of crop evolution [3,28].

Genes involved in the early stage domestication process are key to understanding the domestication process of a crop. Sequence variation from these early stage domestication genes can indicate whether a specific domestication trait had single or multiple causal mutations, revealing whether domestication has a single or multiple origin. The geographic origin and spread of domestication traits can be inferred from sequence variation in domestication loci within contemporary wild and domesticate populations [17,29–32]. In Asian rice, for example, genome-wide single nucleotide polymorphisms (SNPs) have suggested that each rice subpopulation had independent wild rice populations/species as their progenitors [33–37], but the domestication genes revealed a single common origin of these loci [35], suggesting a single *de novo* domestication model for Asian rice [37–42]. On the other hand, the domestication gene for the non-brittle phenotype (*btr1* and *btr2*) in barley had at least two independent origins [43,44], likely from multiple wild or proto-domesticated individuals [45]. This suggests barley follows a multiple domestication model [46–48] originating from multiple ancestral population [45,49,50].

To better understand the domestication of *O*. *glaberrima*, we have re-sequenced whole genomes of *O*. *glaberrima* landraces and its wild progenitor *O*. *barthii* from the hypothesized center of origin in the inner Niger delta, the middle and lower Niger basin that includes the countries Niger and Nigeria, and from Central Africa which includes Chad and Cameroon. The latter two regions were not heavily sampled in previous genomic studies. Together with published *O*. *barthii* samples from West Africa [13] and *O*. *glaberrima* samples from the Senegambia and Guinea region [14], we conducted a population genomic analysis to examine the domestication history of *O*. *glaberrima*. The domestication history were further examined from the evolutionary analysis of genes involved in the early stage domestication process, mainly in the traits involving loss of shattering and erect plant growth. To complement the inferred domestication history, we measured panicle threshability, an important early domestication trait associated with seed non-shattering, from our *O*. *glaberrima* samples to further elucidate the domestication history of *O*. *glaberrima*. With our data we examine the evolutionary and population relationships between *O*. *glaberrima* and *O*. *barthii*, the demographic history, and the geographic origin(s) of domestication of the African rice *O*. *glaberrima*.

## Results and discussion

### Sequence diversity in *O*. *glaberrima* and *O*. *barthii*

We re-sequenced the genomes of 80 *O*. *glaberrima* landraces from a geographic region that spanned the inner Niger delta and lower Niger basin region (S1A Fig). Together with 92 *O*. *glaberrima* genomes that were previously re-sequenced [14], which originated mostly from the coastal region (S1B Fig), the 172 *O*. *glaberrima* genomes analyzed in this study represent a wide geographical range from West and Central Africa. We also re-sequenced the genomes of 16 *O*. *barthii* samples randomly selected from this area, which includes the areas from coastal west Africa, inner Niger delta, and the lower Niger basin (S1C Fig). These were analyzed together with the 94 *O*. *barthii* genomes that were previously re-sequenced [13].

The average genome coverage in the data set we gathered for this study was ~16.5× for both domesticated and wild African rice samples, and is comparable to the sequencing depth (~16.1×) in our previous study. The Wang *et al*. [13] study sequenced a subset of their samples to a higher depth (~19.4×), although the majority of their samples had relatively low coverage (~3.9×) (see S1 Table for genome coverage of all samples in this study). To avoid potential biases in genotyping that arises from differences in genome coverage [51,52], we conducted our population genetic analysis using a complete probabilistic model to account for the uncertainty in genotypes for each individual [53,54]. For the subset of our analysis that required genotype information for each sample, we employed SNPs called from individuals with greater than 10x genome-wide coverage. After quality control filtering, we identified a total of 634,418 and 1,568,868 post-filtered SNPs from the non-repetitive regions of the *O*. *glaberrima* and *O*. *barthii* genomes, respectively.

### Genetic and geographic structure of *O*. *glaberrima*

The genetic structure across domesticated and wild African rice was examined by estimating the ancestry proportions for each individual in our dataset. We employed the program NGSadmix [55], which uses genotype likelihoods from each individual for ancestry estimation and is based on the ADMIXTURE method [56]. Ancestry proportions were estimated by varying the assumed ancestral populations (K) from 2 to 9 groups (S2 Fig).

With K = 2 NGSadmix divided the data set into *O*. *glaberrima* and *O*. *barthii* species, with several *O*. *glaberrima* samples having varying degrees of *O*. *barthii* ancestry (ranging from 4.5 to 40.5%). Interestingly, there were a number of *O*. *barthii* samples that had high proportions of *O*. *glaberrima* ancestry. All these wild rice with discernible *O*. *glaberrima* admixture corresponded to the designated OB-V *O*. *barthii* group and hypothesized progenitor of *O*. *glaberrima* from Wang *et al*. [13]. However, our ancestry analysis suggests this wild *O*. *barthii* group could also be a result of either wild-domesticated rice hybridization or mislabeling of *O*. *glaberrima* as *O*. *barthii* (see below) [57].

**Fig 2:**
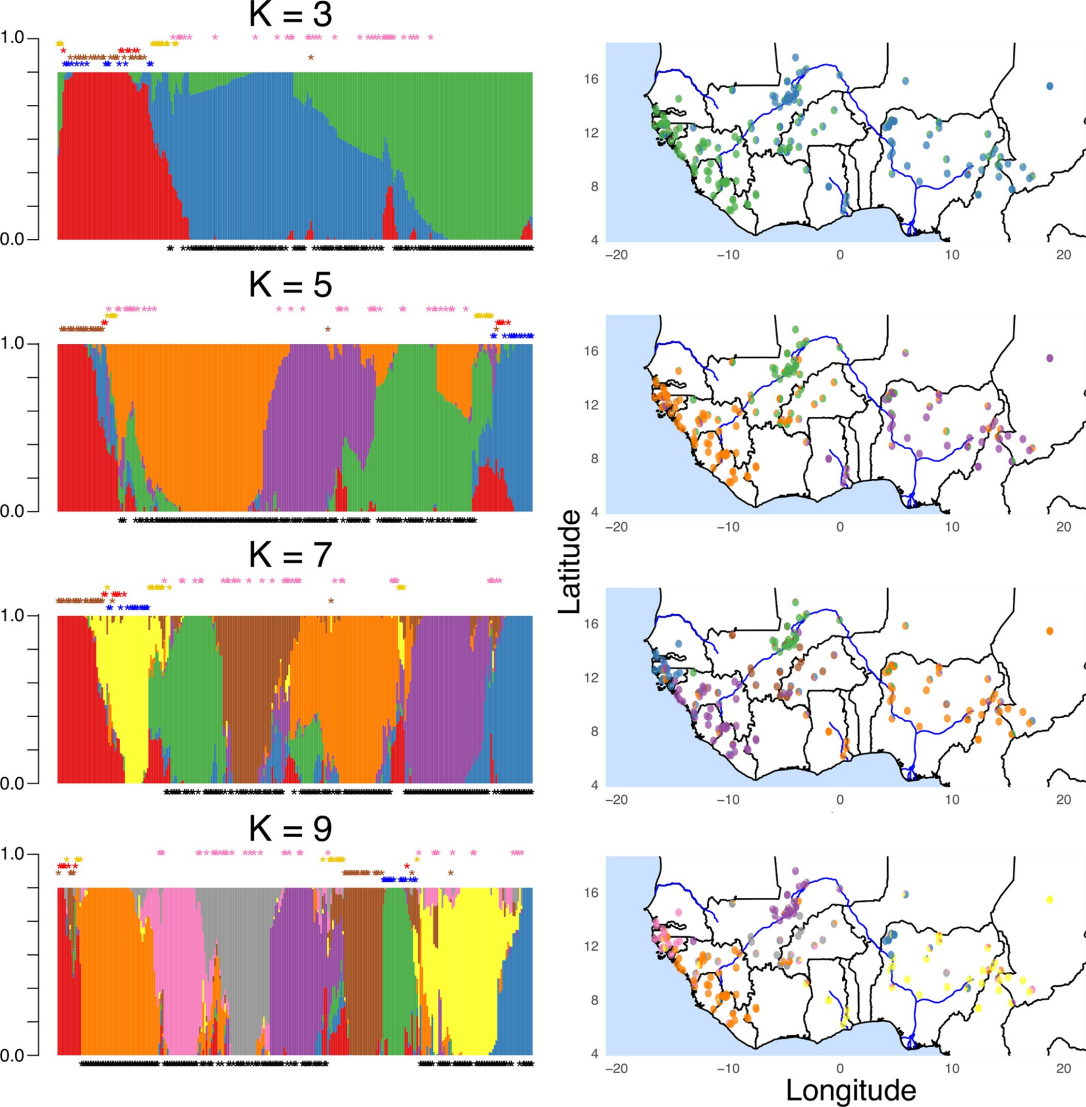
Ancestry proportion estimates and their geographic distribution in *O*. *glaberrima*. On the left panel shows ancestry proportions estimated from NGSadmix assuming K = 3, 5, 7, and 9. Black stars below the admixture barplot indicate *O*. *glaberrima* individuals. Colored stars above admixture barplot are the *O*. *barthii* grouping designated by Wang et al. [13] where blue: OB-I, brown: OB-II, red: OB-III, yellow: OB-IV, and pink: OB-V group. On the right panel shows the ancestry proportion of each individual and their geographical region.

Increasing K further subdivided *O*. *glaberrima* into subpopulations that had a geographical basis (see S2 Fig for all K and their geographic distribution). For instance, K = 3 divided the *O*. *glaberrima* into two major subpopulations, first a coastal population that includes the Senegambia and Guinea highland region, and second an inland population that includes the inland Niger delta and lower Niger river basin region (Fig 2).

At K = 5, there were three major genetic groups within *O*. *glaberrima* and two within *O*. *barthii*. The two *O*. *barthii* genetic groups corresponded to OB-I and OB-II group identified in Wang et al. [13]. For *O*. *glaberrima*, the ancestry proportions showed structuring into 3 major geographic regions: coastal, inner Niger delta, and lower Niger basin populations (Fig 2).

At K = 7, *O*. *glaberrima* were divided into 5 genetic groups where the coastal and inner Niger delta population were further divided into northern and southern genetic groups. It is also at K = 7 where *O*. *glaberrima* divided into genetic groups that are consistent with Portères observation—that the coastal population is divided into a Senegambia or Guinea highland genetic cluster, while the samples closest to the inner Niger delta forms a unique genetic cluster (Fig 2).

At K = 9, *O*. *barthii* is separated into the three genetic groups OB-I, OB-II, and OB-III that were previously identified from Wang et al. [13]. For *O*. *glaberrima* the lower Niger basin population divided into two geographic regions, where the samples closer to the inner Niger delta formed its own genetic cluster (Fig 2). Importantly, what is noticeable with increasing K is that populations appeared to be separating into smaller, and more highly localized geographical clusters.

**Fig 3:**
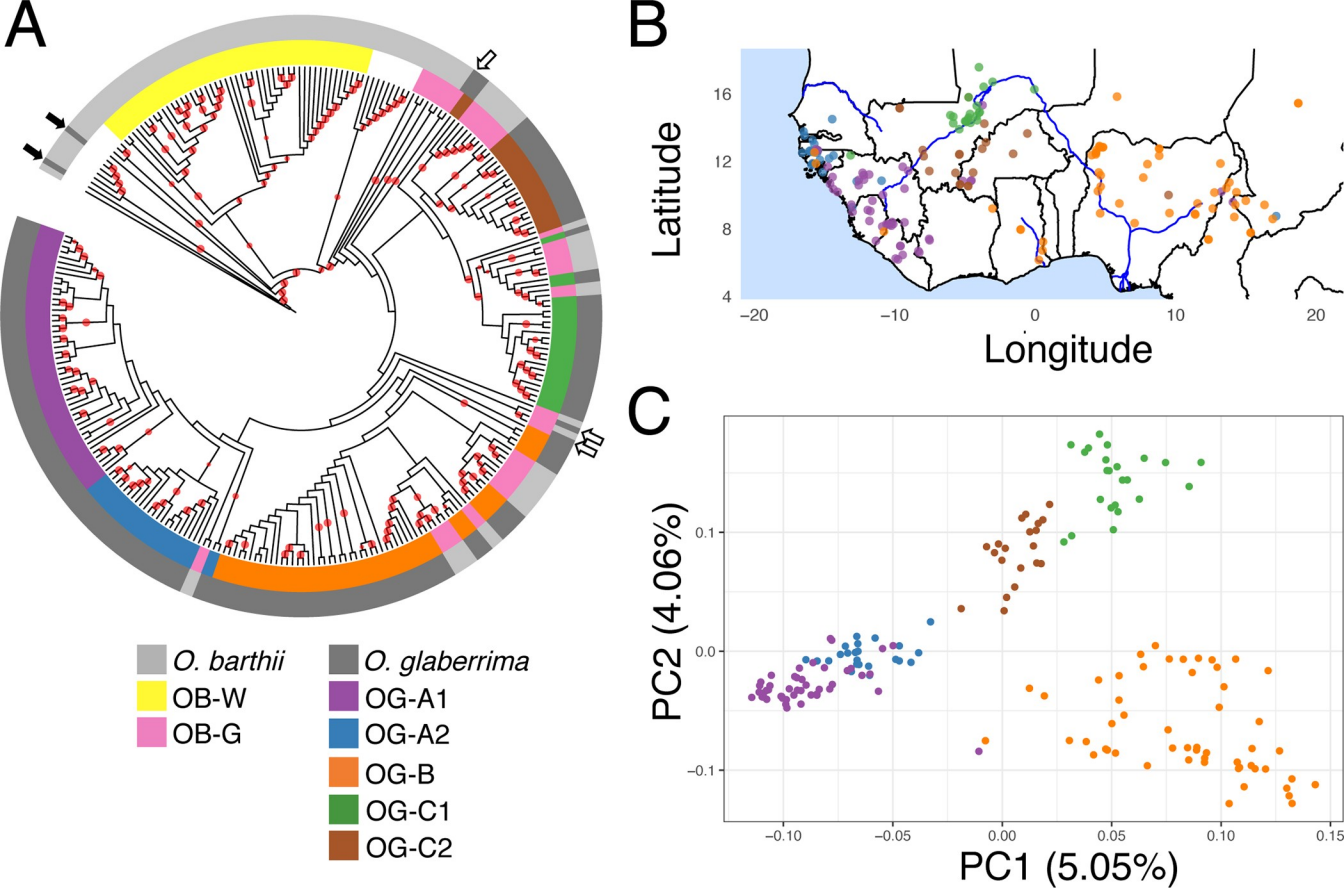
Phylogenomic and principal component analysis of African rice. (A) Neighbor-joining tree built using a distance matrix estimated from NGSdist. Color strips represent group of *O*. *glaberrima* individuals sharing a common ancestor. Internal branches with red circle denote bootstrap support of greater then 80%. Dark arrows indicates the *O*. *glaberrima* grouping with divergent *O*. *barthii* groups, and white arrows indicates the *O*. *glaberrima* grouping with OB-G group *O*. *barthii*. (B) Geographical distribution of each individual and colored by its grouping status as outlined in (A). (C) Principal component analysis conducted using NGScovar. Individuals are color coded according to (A).

We then conducted phylogenomic and principal component analysis (PCA) to verify our ancestry proportion results. Phylogenomic analysis were conducted using genotype likelihoods to estimate the pairwise genetic distances [58] and build a neighbor-joining tree (Fig 3A). *O*. *glaberrima* formed a paraphyletic group relative to several *O*. *barthii* individuals. We noticed that *O*. *glaberrima* landraces could be divided into 5 phylogenetic groups sharing a common ancestral node. Although the bootstrap support on the five ancestral nodes were weak, the geographic distribution of these 5 phylogenetic groups (Fig 3B) were concordant with the geographic distribution of the ancestry components in the *O*. *glaberrima* subpopulations identified at K = 7 (Fig 2, note at K = 7 *O*. *glaberrima* forms five major genetic clusters while *O*. *barthii* forms two major genetic clusters). The 5 phylogenetic groups clustered into five geographic locations: north and south coastal population, north and south inland Niger delta population, and a lower Niger basin population.

The *O*. *glaberrima* population genotype likelihoods were also used for principal component analysis (PCA), which visualized the population relationships [54]. For the PCA plot, individuals were color coded according to the grouping status determined from the phylogenomic results (Fig 3A and Fig 3B). When color-coded according to the phylogenomic tree grouping, PCA results showed 5 independent clusters for *O*. *glaberrima* (Fig 3C). In addition, the distribution of individuals along the two principal components showed striking similarity with their geographic distribution (Fig 3B versus Fig 3C).

Together, our analyses of ancestry components, phylogenomics, and PCA suggest *O*. *glaberrima* has a geographically based population structuring with at least 5 subpopulations (Fig 3A). Consistent with the hypothesized Guinea highland and Senegambia populations, the coastal populations were divided into OG-A1 and OG-A2 genetic groups (collectively the OG-A supergroup). The lower Niger basin and central African individuals formed as a single OG-B group. Finally, for the inner Niger Delta region, landraces closest to the delta formed the OG-C1 group while the others formed the OG-C2 group; collectively these represent the OG-C supergroup.

We note there are several methods of testing and choosing the most appropriate number of genetic clusters (K) for a population sample [56,59–62]. However, these statistical tests can be misleading [63,64], often prompting overconfidence in a single K value that may or may not be biologically relevant. Thus, we emphasize our choice of dividing *O*. *glaberrima* into five major groups represent the minimum possible grouping based on historical observations [8,9] and geography (Figs 2 and 3). We also find that these 5 groups had significant correlations with the geographical distributions of domestication gene mutations and phenotypes (see below domestication gene analysis for more detail), further suggesting they represent biologically relevant groupings.

### Genetic structure of *O*. *barthii*

At K = 7 the majority of the newly sequenced *O*. *barthii* from this study belonged to either OB-I or OB-II subpopulations designated by Wang *et al*. [13] (S3 Fig). The ancestry proportion for the OB-III and OB-IV groups suggested these individuals were an admixed group, with OB-III an admixture of OB-I and OB-II, and the OB-IV group possessing a mix of ancestry from both wild and domesticated rice. Note that at higher K values, OB-III formed its own genetic cluster while OB-IV showed ancestry with large proportions from wild and domesticated rice. Unlike the OB-V group of *O*. *barthii*, which also had several individuals of mixed wild and domesticated rice ancestry, the OB-IV group did not phylogenetically cluster with *O*. *glaberrima* (Fig 3 and S3 Fig). This suggests that the OB-IV subpopulation may be an evolutionary distinct population, and the ancestry proportions were possibly mis-specified [64]. Hence, we considered individuals that were monophyletic with the OB-I or OB-II subpopulations as the wild *O*. *barthii* subpopulation and henceforth designated it as OB-W [= wild] (Fig 3A). *O*. *barthii* that were paraphyletic with *O*. *glaberrima* were considered as a separate *O*. *barthii* group and designated as OB-G [= *glaberrima*-like] (Fig 3A). Geographically, OB-G was found throughout West Africa but OB-W was found mostly in inland West African countries such as in Mali, Cameroon, and Chad (S2 Table).

### Relationships between *O*. *glaberrima* populations

Before examining the relationships between our 5 inferred genetic clusters for *O*. *glaberrima*, we filtered individuals with spurious classification.

First, we find that there were 3 *O*. *glaberrima* individuals (IRGC104883, IRGC105038, and IRGC75618) that did not group with any of the 5 population groups (Fig 3A white arrow), but rather formed as a sister group to all *O*. *glaberrima* or sister group to both OG-A and OG-B group. Because they were most closely related to OB-G samples we considered them as OB-G as well. Interestingly, there were also two *O*. *glaberrima* individuals (IRGC103631 and IRGC103638) that phylogenetically clustered with *O*. *barthii* (Fig 3A filled arrows). Ancestry estimates for the two samples showed high proportions of both *O*. *glaberrima* and *O*. *barthii* ancestry (S4 Fig). These two *O*. *glaberrima* samples were not used in subsequent analyses. Moreover, all *O*. *barthii* samples not grouped as OB-W or OB-G, as discussed above, were excluded from downstream analysis.

Second, we examined other potentially spuriously grouped individuals by calculating silhouette scores for each individual [65], which measures similarity with members of its own group compared to members of other groups (see Materials and Method for details). Initially, 174 *O*. *glaberrima* samples with greater then 10× coverage were used for genotyping. A multidimensional scaling (MDS) plot of the population and their grouping status showed that even before the silhouette score-based filtering, there were clear separation among the OG-A, OG-B, and OG-C groups (S5A Fig). But there were also several individuals whose status was questionable, as they overlapped in coordinate space with other groups. Individuals with negative silhouette scores (i.e. potential mis-grouping) or scores lower then 0.12 (i.e. individuals with significant portions of ancestry coming from a different genetic group) were filtered out (S5B Fig) to remove individuals with questionable grouping status and thus specify genetically unique populations [66]. We note some individuals that were filtered from the silhouette score-based method were likely filtered because they are admixed individuals. Omission of those individuals would lead to an underestimation of the recent admixture history of *O*. *glaberrima*. Here, our interest is in determining the long-term population histories that shaped each *O*. *glaberrima* population; hence, removal of those recently admixed individuals are necessary.

This last filtering process resulted in 94 individuals, which we refer to as the core set population (see S3 Table for list of accessions). MDS plot of this core set population showed clear separation among each other (S5C Fig), suggesting these are genetically distinct populations (S5D Fig). This core set population was used to infer the population relationship within *O*. *glaberrima*. To determine the population relationships, we also included polymorphism data from the OB-W group individuals with greater then 10× coverage. Because our grouping is based on K = 7 ancestry (Fig 2), which had two population groups for OB-W, we divided the OB-W group into two (OB-W1 and OB-W2) based on the common ancestor they shared in the phylogenomic tree (Fig 3A). For an outgroup population, polymorphism data from six *O*. *rufipogon* individuals with greater then 10× coverage were used [35]. An MDS plot of the nucleotide variation showed clear separation among the three species and separation within species depending on the population grouping status (S6 Fig).

**Fig 4:**
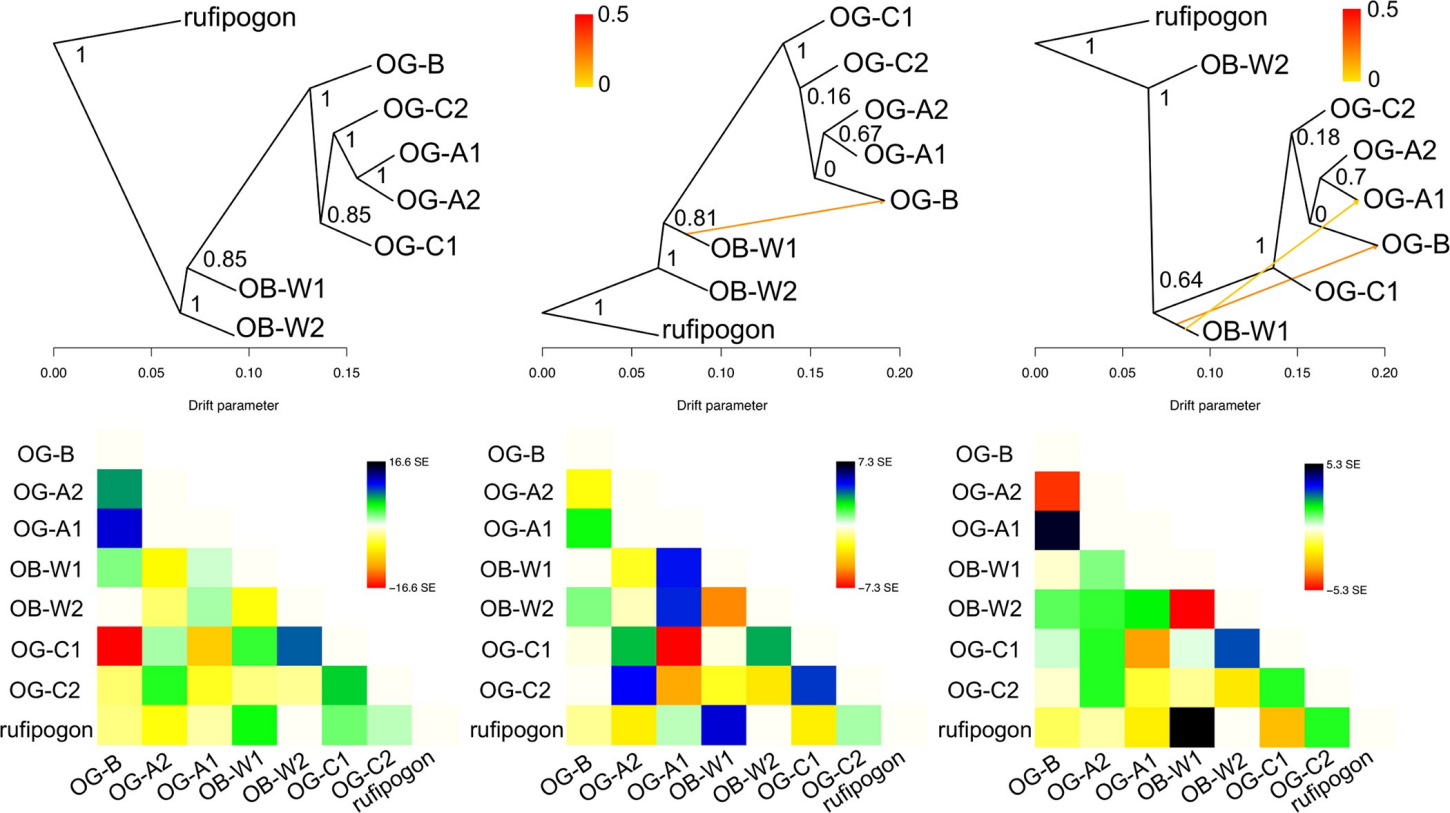
Treemix admixture graph of genetic groups from *O*. *barthii*, *O*. *glaberrima*, and *O*. *rufipogon*. Admixture graphs are shown for models assuming zero to two migration events. Numbers on nodes represent bootstrap support after 100 replicates. Residuals for each migration model are shown below the graph.

Using the core set population, we inferred the population relationships between the five genetic groups of *O*. *glaberrima* with Treemix [67]. For the graph rooted with *O*. *rufipogon* population, without modeling any migration events the OB-W1 group were sister to all *O*. *glaberrima* (Fig 4). This model without any migration events was able to explain 99.4% of the variance, suggesting most of the allele frequency variability in the data can be explained without evoking migration between groups. Nevertheless, residuals from the covariance matrix suggested several population pairs could be more closely related (population pairs with positive residuals) compared to the best-fitting tree.

Fitting models with 1, 2, and 3 migration events brought marginal increase in the variance explained for each migration model (variance increases as 99.8%, 99.9%, and 99.94% respectively). Fitting 1 and 2 migration events suggested an admixture event between a population ancestral to OB-W1 and modern OG-A1 or OG-B (Fig 4). This suggests an unsampled *O*. *barthii* population may have admixed with OG-A1 and/or OG-B population. The first within-*O*. *glaberrima* admixture, specifically between OG-A1 and OG-B, was observed in the model fitting 3 migration events (S7 Fig). But the *f4* test [68] indicated no significant deviation from non-admixed topology for the tree [[wild rice, OG-B],[OG-A1,OG-A2]], suggesting the 3 migration model is an overfitted model (see S4 Table for *f4* test result).

Collectively, our analysis suggests the *O*. *glaberrima* population could be modeled as a bifurcating tree-like population, with small ancient admixture events from *O*. *barthii* genetic groups. Here then, it is tempting to interpret the *O*. *glaberrima* population topology, specifically the order of splitting of each genetic group, as the order of the domestication/diversification events. However, we should note that the topology changes with and without modeling migration, and in higher migration models several population pairs (e.g. based on the residuals between OG-C1 and OB-W2) are still not well fitted, while bootstrap support for several internal branches are low. Thus, while this analysis provides an initial framework for depicting population relationships, one should exercise caution in over-interpreting the inferred trees.

### The OB-G group of *O*. *barthii* is not the direct progenitor of African rice

Previous molecular studies have argued the close genetic affinities of some west African *O*. *barthii* (namely the OB-G group in this study) to *O*. *glaberrima*, as evidence of the former being the progenitor population of African rice [13,69]. We thus examined the properties of the OB-G group in relation to OB-W and *O*. *glaberrima*.

**Fig 5:**
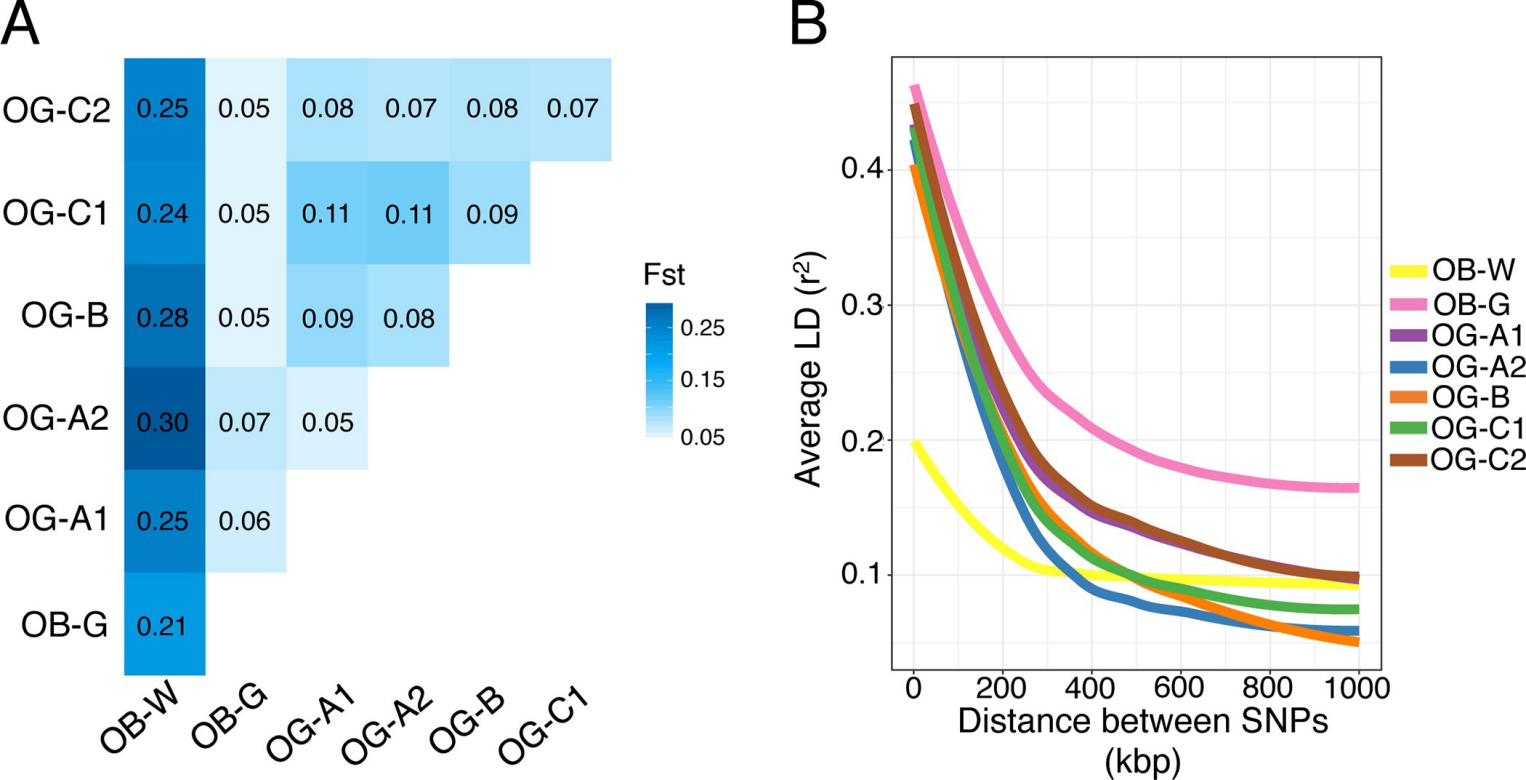
Evolutionary relationship between wild and domesticated African rice. (A) Pairwise Fst values between *O*. *glaberrima* and *O*. *barthii* genetic groups. (B) Average linkage disequilibrium between pair of SNPs.

First, we found that the level of population differentiation between OB-G and *O*. *glaberrima* was low (~ 0.06) (Fig 5A), almost comparable to the level seen between *O*. *glaberrima* genetic groups (~0.09). In contrast, there is a higher level of differentiation between each *O*. *glaberrima* genetic group and OB-W (Fst ~ 0.26). Similarly, OB-G group also had high levels of differentiation to OB-W (Fst ~ 0.21).

Second, we examined levels of linkage disequilibrium (LD) decay, as wild and domesticated populations have different LD profiles, due to the latter undergoing domestication-related bottlenecks and selective sweeps [70]. In the African rice group, as expected, all *O*. *glaberrima* genetic groups had higher levels of LD compared to the OB-W group (Fig 5B). The OB-G group also had high levels of LD that was comparable to those observed in *O*. *glaberrima*, although compared to other OG groups, the OB-G group had longer tracts of LD.

Finally, genome-wide polymorphism levels for the OB-G group were also comparable between OB-G and *O*. *glaberrima*. Specifically, compared to the OB-W group, SNP levels and Tajima’s D [71] were significantly lower in both OB-G and *O*. *glaberrima* (S8 Fig).

Together, the levels of genetic differentiation, linkage disequilibrium, SNP levels and patterns, all suggest that the OB-G genomes resemble *O*. *glaberrima* more than *O*. *barthii*. Furthermore, the majority of the OB-G samples carried at least one domestication mutation (see domestication gene haplotype analysis section for detail), calling into question its status as the wild progenitor. In contrast all OB-W individuals do not carry the causal mutation/deletion at known domestication genes. All in all, this suggests the OB-G population is actually *O*. *glaberrima* that was mislabeled as *O*. *barthii*. It is also possible that this population may represent feral weedy rice [72], resulting from the hybridization of domesticated and wild African rice; this is certainly consistent with the increased LD structure within OB-G [73]. While the different demographic histories between the source populations can generate an overall negative Tajima’s D for the resulting admixutre population [74]. Together, our results suggest that OB-G may have formed after the domestication event and supports a de-domestication (endoferality) origin for that group [57].

### *PROG1* is deleted in all *O*. *glaberrima* and likely originated from central Africa

To further identify the domestication origin(s) of *O*. *glaberrima*, we examined the haplotypes for the domestication genes involved in erect plant growth (*PROG1*) and the non-shattering phenotype (*sh4* and *sh1*) in both wild and domesticated African rice. We took an approach we term *functional phylogeography*, where we examined the haplotype structure surrounding the domestication gene of interest [29], inferred a haplotype phylogenetic network, and determined the geographic origin and spread of the functional mutation by comparing the geographic distributions of haplotypes in wild and domesticated African rice in a phylogenetic context. Because we focused on the non-recombining region surrounding a domestication gene, there were only a few sites being analyzed between *O*. *glaberrima* and *O*. *barthii*. However, we were specifically interested in those few mutations that differ between the domestication gene haplotype and the progenitor gene haplotype, and used those differences to build the haplotype phylogenetic network.

The *PROG1* gene was first identified as a domestication gene in the Asian rice *O*. *sativa*. A mutation in this gene causes the plant to grow erect in both Asian and African rice, increasing growing density and enhancing photosynthesis efficiency for higher grain yields [19,20,75,76]. Our analysis of *O*. *sativa PROG1* gene orthologs in *O*. *glaberrima* and *O*. *barthii* indicates that this gene is missing only in *O*. *glaberrima* (S5 Table). We expanded the analysis to our population dataset, and a sequencing read depth analysis found *PROG1* was missing in all *O*. *glaberrima* landraces (Table 1). None of the OB-W individuals had the *PROG1* deletion and all but two of the OB-G individuals had the *PROG1* deletion. Synteny of the genes immediately surrounding *O*. *sativa PROG1* was maintained in both *O*. *glaberrima* and *O*. *barthii*, suggesting the *PROG1* gene is deleted specifically in *O*. *glaberrima*. Because of its importance in early domestication and lack of gene structure in *O*. *glaberrima*, we considered *PROG1* as a candidate domestication gene in African rice and examined the population genetics of the *PROG1* gene in *O*. *glaberrima* and *O*. *barthii*. We note this is the first candidate domestication gene that has been identified where the causal mutation is fixed in all *O*. *glaberrima* population.

**Fig 6:**
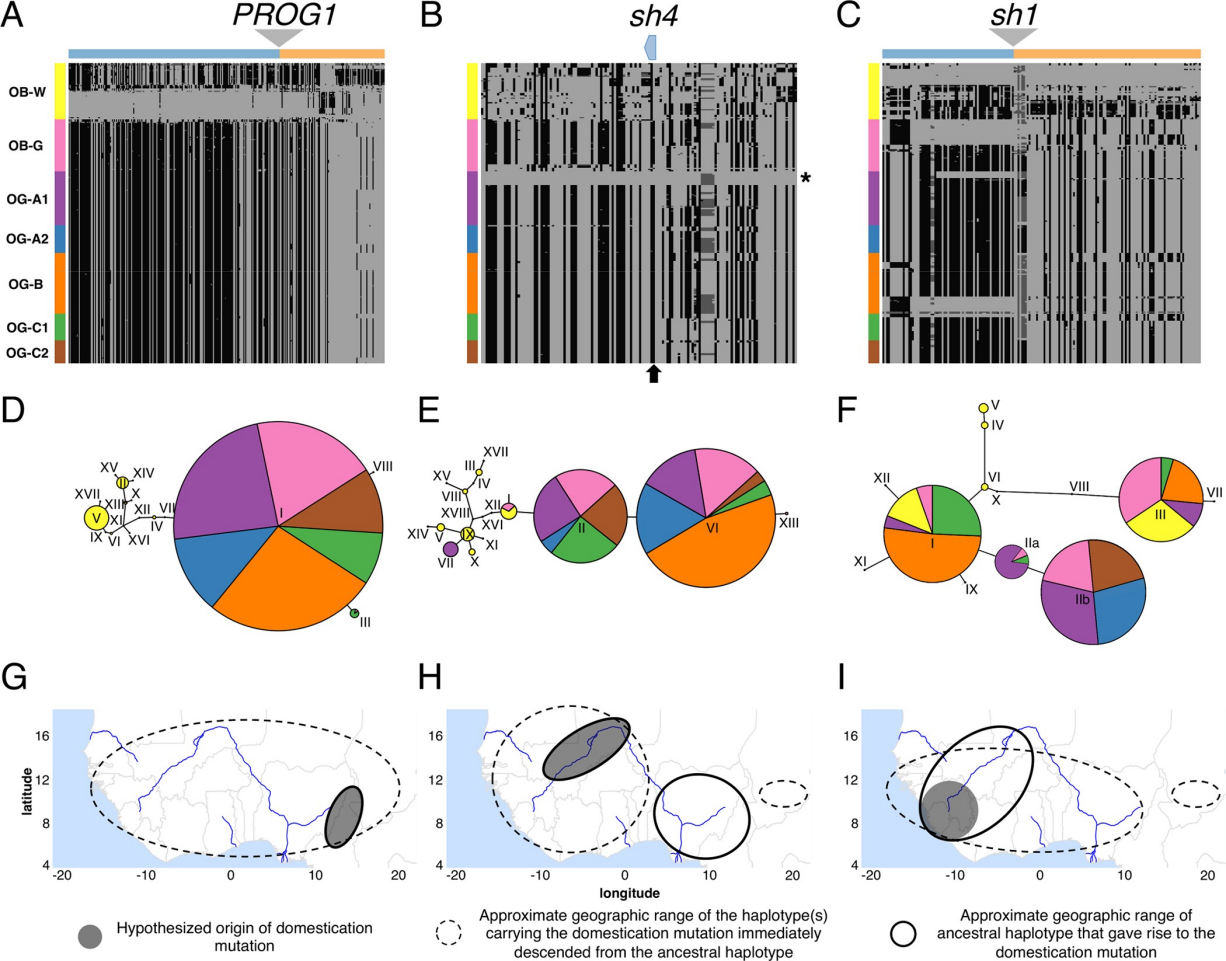
**Haplotype analysis of the three domestication genes (A,D,G) *PROG1*, (B,E,H) *sh4*, and (C,F,I) *sh1*.** Haplotype structures are shown for the genes (A) *PROG1*, (B) *sh4*, and (C) *sh1*. Homozygote genotype not identical to reference genome is shown in dark grey, heterozygote genotype shown in lighter shade of grey, and homozygote genotype identical to reference genome shown in lightest shade of grey. Regions are showing polymorphic sites from 25 kbp up- and downstream of the domestication gene. In *sh4* (B) the position of the causal domestication mutation is shown in arrow and OG-A1 samples without the causal mutation are indicated with a star. In *PROG1* (A) and *sh1* (C) region upstream and downstream the deletion are color coded above the haplotype structure. Haplotype network are shown for genes (D) *PROG1*, (E) *sh4*, and (F) *sh1*. Approximate geographic origins for the causal domestication mutation haplotype and its most closely related ancestral haplotype are shown for genes (G) *PROG1*, (H) *sh4*, and (I) *sh1*. See text for discussion of the hypothesized geographic origins.

**Table 1:** Domestication allele status in *O*. *glaberrima* and *O*. *barthii* groups.

	*sh4*		*sh1*		*PROG1*	
Genetic Group	Causal*	Other	*sh1Δ*	*sh1*	*PROG1Δ*	*PROG1*
OG-A1	35	12	15	32	47	0
OG-A2	24	0	24	0	24	0
OG-B	53	0	0	53	53	0
OG-C1	23	0	0	23	23	0
OG-C2	20	0	20	0	20	0
OB-G	39	0	15	30	43	2
OB-W	NA		0	49	0	49

*For *sh4* gene, samples were divided depending on whether they carried the haplotype with the nonsense mutation (Causal) or not (Other). OB-G individuals that were similar but did not carry the nonsense mutation (haplotype I in Fig 6E) were classified into the Causal group.

We first examined whether the *PROG1* locus showed evidence for positive selection in *O*. *glaberrima*, using genome-wide sliding window analysis of the ratio of polymorphism between the wild OB-W group to all domesticated *O*. *glaberrima* (π_w_/ π_D_). A domestication-mediated selective sweep would lead to a reduction in nucleotide variation around the target domestication gene, but only within the domesticated group. Because *PROG1* is deleted in *O*. *glaberrima*, the selection signal will only persist around the candidate deletion region. Spanning 10 kbp of the candidate deletion region, π_w_/ π_D_ is within the top 1% value, and this is observed regardless of whether the *O*. *glaberrima* or *O*. *barthii* genome was used as the reference genome in SNP calling (S9 Fig). This is consistent with the *PROG1* region having gone through a selective sweep during *O*. *glaberrima* domestication. Cubry et al. [15] has also independently found evidence of a selective sweep in the *PROG1* region of *O*. *glaberrima*, supporting our finding that this region has been a target of domestication-related selection.

Polymorphisms surrounding the *PROG1* deletion comprised a single unique haplotype segregating across all *O*. *glaberrima* samples and most of the OB-G samples (Fig 6A). A haplotype network of a non-recombining 5 kbp region immediately upstream of the deletion showed that all individuals with the deletion belonged to the same major haplotype group, with the dominant haplotype I, as well as peripheral haplotypes III, VII, and VIII (Fig 6D). Maximum-likelihood tree of the region surrounding *PROG1* collapsed all *O*. *glaberrima* into a single phylogenetic group (S10A Fig), which suggests a single origin for the deletion. We tabulated the geographic distributions of *PROG1* haplotypes (S6 Table). *PROG1* haplotype VII is the earliest haplotype with the deletion and is found in an OB-G individual from Cameroon. The ancestral non-deleted *PROG1* haplotype was carried by haplogroup IV (Fig 6D), which was most closely related to all haplotypes with the *PROG1* deletion, and was made up of three OB-W individuals: IRGC103912 (Tanzania), IRGC105988 (Cameroon), and WAB0028882 (Cameroon). The downstream region of the deletion was consistent with what is observed in the upstream region (S11 Fig). Twenty-two polymorphic sites from a non-recombining 7 kbp downstream region show the same OB-W individuals (IRGC105988 and WAB0028882), both from Cameroon, were the most closely related haplotype to the *PROG1* deletion haplotype. Maximum-likelihood trees of both the upstream and downstream regions also showed these two individuals to be the sister group to all *O*. *glaberrima* samples (S10A Fig).

Together, the geographic distribution of the *PROG1* region haplotypes suggest that the *PROG1* deletion may have occurred in a wild progenitor closely related to those found in Cameroon, Central Africa, and spread throughout West Africa to the different *O*. *glaberrima* genetic groups (Fig 6G). The *PROG1* conclusion must be tempered, however, by an acknowledgment that the sample size of ancestral haplotypes is small (n = 3). Interestingly, a similar observation has been made in *O*. *sativa* where all Asian rice subpopulations are monophyletic in the *PROG1* region, but genome-wide the different Asian rice variety groups/subspecies do not share immediate common ancestors [35,77].

### The geographic origin of the *sh4* nonsense allele

Evidence for a selective sweep around the causal domestication mutation, a C-to-T nonsense mutation at position 25,152,034 leading to a loss-of-function allele (Fig 6B arrow), has been previously shown [78,79] for the *sh4* gene (*O*. *glaberrima* chromosome 4:25,150,788–25,152,622). The haplotype structure around the *sh4* gene showed most of the *O*. *glaberrima* landraces carried the causal domestication mutation (Fig 6B). Several individuals within OG-A1 group, including the reference genome, did not carry the causal mutation but still had long tracks of homozygosity at the *sh4* locus (Fig 6B star).

A four-gamete test [80] of the 4 kbp upstream and 2 kbp downstream region spanning the *sh4* gene detected evidence of recombination, within the *O*. *barthii* population (both OB-G and OB-W) and but not within *O*. *glaberrima*. A maximum-likelihood tree of the region surrounding *sh4* showed all *O*. *glaberrima* populations were divided into two major phylogenetic groups, but with weak bootstrap support (S10B Fig). *O*. *glaberrima* individuals without the causal mutation (Fig 6B star) formed their own phylogenetic group (S10B Fig star).

To determine the origin of the non-shattering trait, we reconstructed the haplotype network of the non-recombining region of the *sh4* gene in all *O*. *glaberrima* and *O*. *barthii* genetic groups (Fig 6E). Majority of the *O*. *glaberrima* and OB-G group *sh4* haplotypes belonged to haplotypes II, VI, and XIII and they all shared the nonsense mutation. The two main haplotypes II and VI corresponds to the difference observed in the upstream region of the *sh4* gene (Fig 6B), with haplotype II arising prior to haplotype VI. The closest haplotype to II was haplotype I, which was separated by two mutations (position 25,146,871 and the causal domestication mutation 25,152,034).

**Fig 7:**
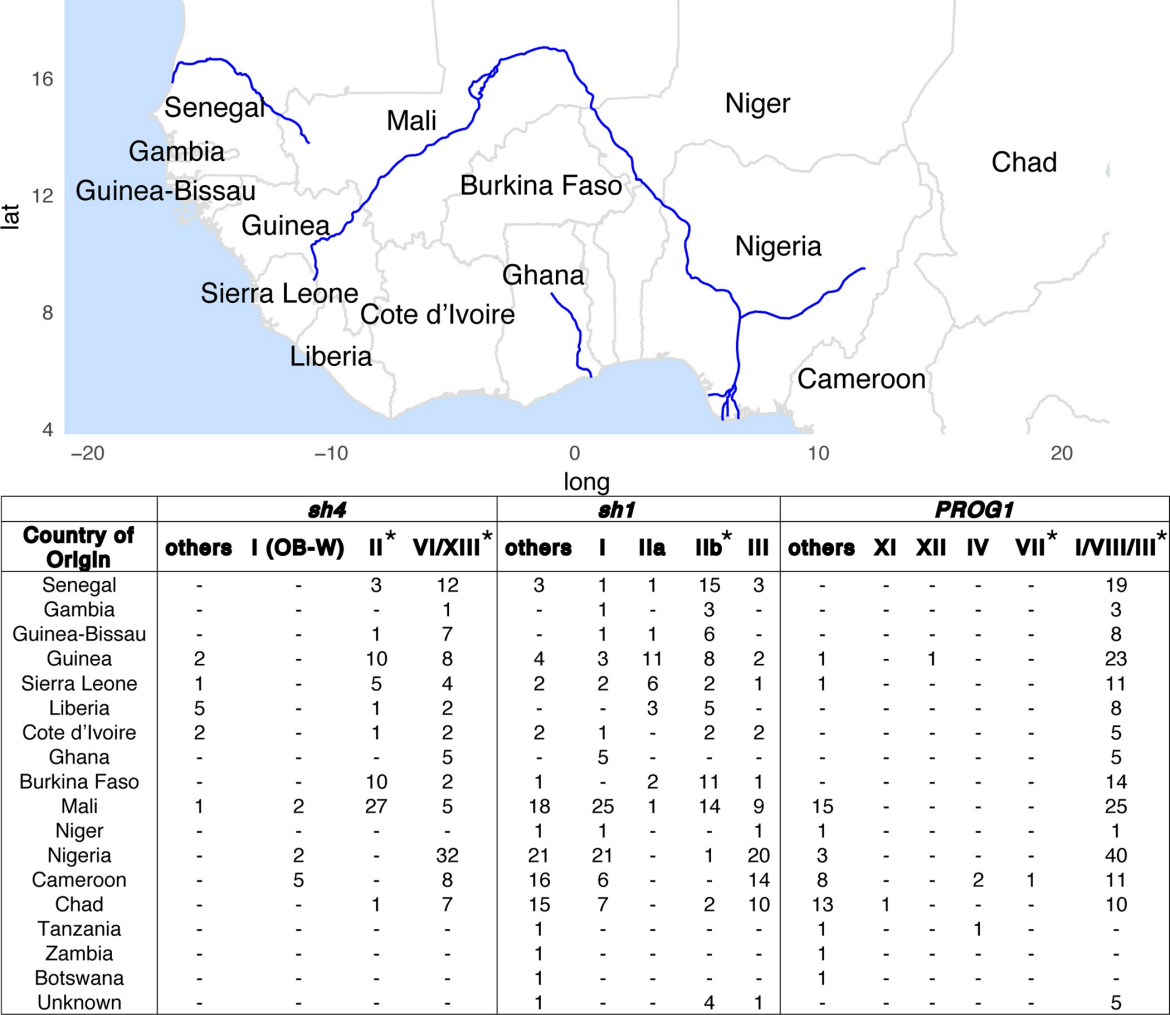
Geographical origin of the domestication gene haplotypes. Haplotypes carrying the domestication mutation are indicated with a star (*). For haplotypes without the casual domestication mutation, only those that are most closely related to the haplotype with the causal mutation are shown. Haplotype numbers and their corresponding relationships are shown in Fig 6.

We tabulated the geographic distributions of *O*. *glaberrima* haplotypes II and VI/XIII, and haplotype I from the *O*. *barthii* OB-W group (Fig 7). The ancestral haplotype I is found in 13 *O*. *barthii* individuals (4 OB-G group and 9 OB-W group), and these individuals originated over a wide geographic region of West Africa that includes both coastal and inland areas (See S8 Table for full list of members of each haplogroup and their country of origin). Of those in OB-W, 2 are from Mali, 2 from Nigeria and 5 are from Cameroon. Among the *O*. *glaberrima* that have the *sh4* mutation, the older haplotype II is found mostly in Mali, Burkina Faso and also Guinea.

Here, we made the assumption that the areas of overlap between the ancestral haplotype (without the causal mutation) and the derived haplotype (with the causal mutation) is likely the place of origin of the domestication allele. For *sh4*, the distribution of haplotype II overlaps with haplotype I in Mali, pointing to Mali as being a likely place of origin for the *sh4* nonsense mutation (Fig 6H). The haplotypes VI and XIII thus subsequently evolved from haplotype II, which expanded over a much wider area, particularly in the Senegambia, and also to Nigeria, Cameroon and Chad. It should be noted that the sample size for haplotype I among OB-W is relatively small (n = 9) leading to disjoint geographic ranges for its distribution (Fig 6H). Localizing the origin of the *sh4* causal mutation to Mali may be revised as more *O*. *barthii* samples are analyzed. However, haplotype II is found at highest frequency in Mali as well (~46%, see Fig 7), which provides further support for a Malian origin of the *sh4* mutations.

Wu *et al*. [79] had first noticed that several *O*. *glaberrima* individuals in the coastal region of West Africa did not have the causal domestication mutation in the *sh4* gene (Fig 6B star). Our data shows that all inland *O*. *glaberrima* carries the haplotype with the nonsense mutation, and the haplotype without the nonsense mutation was indeed limited to the coastal region, specifically in the OG-A1 genetic group. The haplotype network and neighbor-joining tree suggests these individuals had distinct evolutionary histories for the *sh4* gene (Fig 6E and S10B Fig); they carry haplotype VII which is confined to Guinea. The non-fixed status of the nonsense mutation suggests a role of independent mutation(s) in domestication for non-shattering in haplotype VII carriers.

#### The *sh1* gene deletion is polymorphic and has coastal origins

Assembly of the reference *O*. *glaberrima* genome had first shown the gene *sh1* was missing in the *O*. *glaberrima* genome but not in the *O*. *barthii* genome [13]. Recently, the *sh1* gene (see Materials and Method section “Shattering gene nomenclature” for comment on gene nomenclature of *sh1*) was identified as another causal gene for the non-shattering trait, and the causal mutation was indeed a gene deletion that was polymorphic in several coastal *O*. *glaberrima* populations [81]. A read-depth based analysis (see Materials and Method section for details) showed the *sh1* gene was missing in several *O*. *glaberrima* individuals (Table 1). Specifically, no individuals in the inland genetic groups OG-B and OG-C1 had the *sh1* deletion, but in another inland genetic group OG-C2 all individuals carried the *sh1* deletion. In the coastal population all individuals from the Senegambia genetic group OG-A2 had the *sh1* deletion, while in the genetic group OG-A1 the deletion was polymorphic (frequency ~ 32%). The deletion was also polymorphic in the OB-G group (frequency ~ 33%) but no individual had a deletion in the OB-W wild group. Spanning 10 kbp of the *sh1* gene region, *O*. *glaberrima* samples with the *sh1* gene deletion had π_w_/ π_D_ values within the top 1% value while *O*. *glaberrima* samples without the deletion did not show the decreased polymorphism (S12 Fig). This suggested the *sh1* deletion had also undergone a domestication-related selective sweep.

Reflecting the polymorphic status of the *sh1* deletion, we observed no single haplotype at high frequency (Fig 6C). With evidence of recombination in the downstream 5 kbp of the deletion in both wild and domesticated African rice, an unambiguous haplotype network could not be inferred for that region. The haplotype network of a non-recombining 5 kbp region immediately upstream of the deletion with 22 polymorphic sites, indicated that there were 3 main *O*. *glaberrima sh1* haplotypes–I, II and III (Fig 6F). All *O*. *glaberrima* in haplotypes I and III do not carry the *sh1* deletion (See S9 Table for full list of members of each haplogroup and their country of origin). Haplotype II can be further divided into two depending on the status of the *sh1* gene deletion. Haplotype IIb contains all of the *O*. *glaberrima* individuals with the *sh1* deletion, while haplotype IIa is found in 23 OG-A1, 1 OB-G, and 1 OG-C1 individuals and does not carry the *sh1* deletion. A maximum-likelihood tree of the region surrounding *sh1* confirmed the haplotype network, where all OG-A2 and OG-C2 individuals that had the deletion grouped together with several OG-A1 individuals (S10C Fig).

Together, these results indicate that the *sh1* deletion might have arisen on a genetic background most closely related to the OG-A1 genetic group, which in turn suggests a coastal origin for the *sh1* deletion. This coastal origin is supported by the geographic distributions of the different *sh1* haplotypes. Haplotype IIb, which contains the *sh1* deletion, is found across a wide area in West and Central Africa (Fig 7). Haplotype IIa, which does not have the deletion and is presumably ancestral to IIb, is found at highest frequency in Guinea and Sierra Leone. Given where the distributions of haplotypes IIa and b overlap, it would suggest that a region encompassing the coastal countries of Guinea and Sierra Leone was where the *sh1* deletion originated (Fig 6I).

Another study [81] had found the *sh4* and *sh1* double mutant was most prevalent in *O*. *glaberrima* individuals from Senegambia (identified here as the OG-A2 genetic group). Here, we corroborate their findings and further identify that the double mutant was also selected in the inland region but only in the OG-C2 genetic group (Table 1), which is found in southern Mali and Burkina Faso region (Fig 2). It is unclear why the double mutant had not spread further inland or existed in a polymorphic state in the OG-A1 genetic group, but aspects of this behavior is also seen in *O*. *sativa* where the causal mutation in the *qSh1* gene, which produces the non-shattering phenotype, is found only in the temperate japonica subpopulation [18]. Non-shattering is often considered as the trait selected in the earliest stages of rice domestication; however the process may have continued well into the diversification/improvement phase as well. In *O*. *glaberrima*, *sh1* and *sh4* double mutant causes a complete ablation of the abscission layer leading to a complete non-shattering phenotype [81], which would have led to a higher yield per plant but at the cost of increased labor for separating the grains off the rachis [82]. The cost involved in non-shattering may have led to different preferences for the trade-off between harvesting and threshing among the people cultivating *O*. *glaberrima*, which could account for the polymorphism in the frequency of the mutation conferring non-shattering in both *sh4* and *sh1* genes.

### *O*. *glaberrima* panicle threshability is geographically and genetically structured

Our results showed the causal domestication mutations for the shattering genes *sh1* and *sh4* were not fixed in several *O*. *glaberrima* varities, suggesting their seed non-shattering may be incomplete. Thus we examined the phenotypic consequence of the domestication-related selection process of non-shattering by measuring panicle threshability for *O*. *glaberrima*.

We measured the degree of non-shattering in 149 *O*. *glaberrima* accessions according to the Standard Evaluation System for Rice (SES) [83]. We report our measurement of panicle threshability, which is directly related to seed shattering, on a scale of 1, 3, 5, 7, and 9, which indicates a percent shattering of less than 1%, 1–5%, 6–15%, 26–50%, and 51–100% respectively (see S10 Table for each *O*. *glaberrima* individuals’ shattering score).

**Fig 8:**
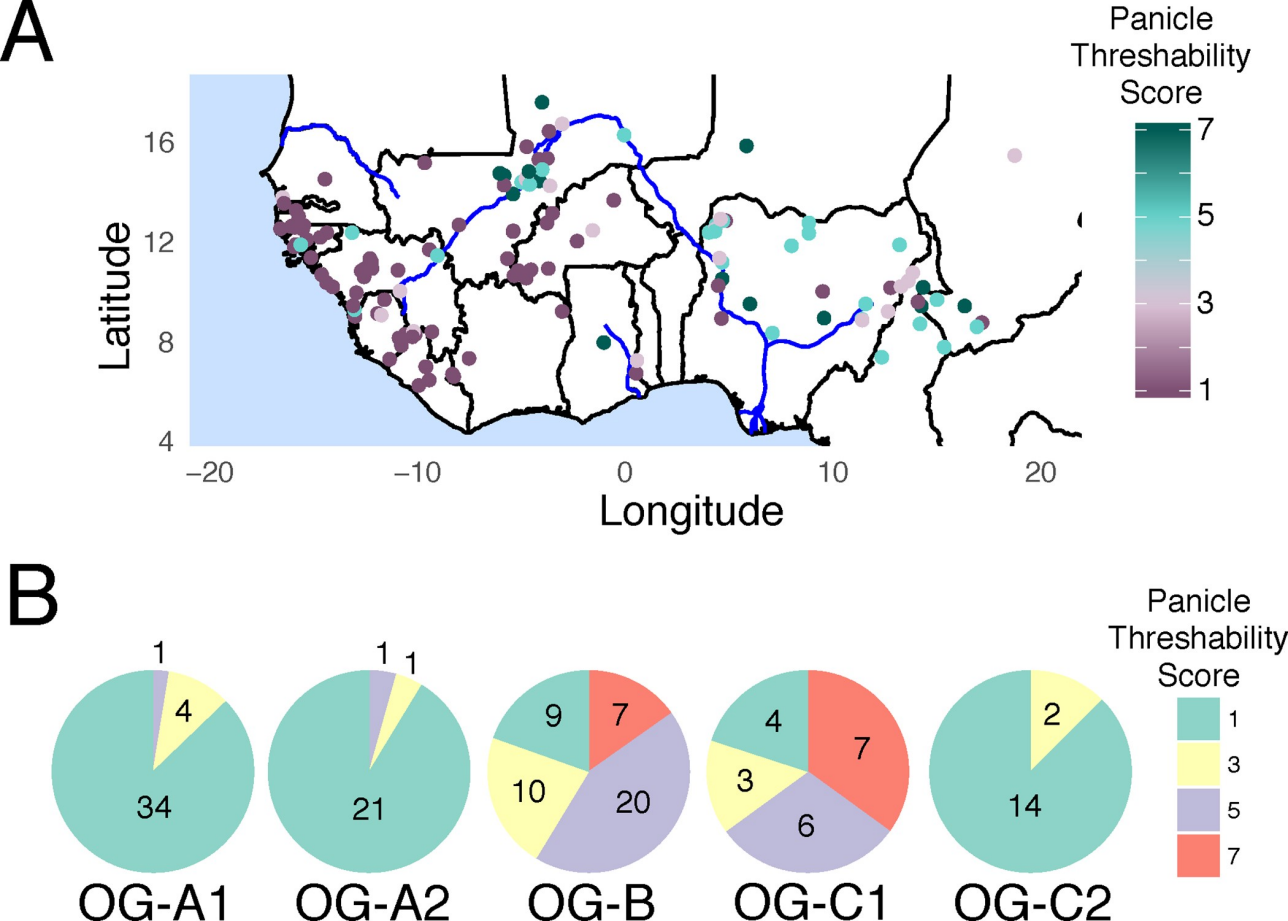
Panicle threshability scores in *O*. *glaberrima*. (A) Geographical distribution of the panicle threshability scores. (B) Pie chart showing the number of individuals and their panicle threshability scores across the 5 genetic groups designated from this study.

The geographic distribution of the panicle threshability score showed an east to west gradient, where inland *O*. *glaberrima* varieties were more likely to have samples with higher threshability score values (Fig 8A). Specifically, the OG-B and OG-C1 group had a mix of individuals with varying degree of shattering, while the groups closer to the coastal area, namely the OG-A1, OG-A2, and OG-C2 group, had predominantly individuals with panicles that were non-shattering (Fig 8B). We compared the shattering scores for each genetic group by conducting Mann-Whitney U test for all pairwise combinations (S11 Table). Results showed significant difference in shattering scores between the coastal and inland genetic groups (OG-B and OG-C1 vs. OG-A1, OG-A2, and OG-C2).

Noticeably the threshability scores were consistent with the shattering mutation results (Table 1). In the coastal region, most individuals (with the exception of the OG-A1 group and see below for detail) had both the *sh1* and *sh4* mutations and were non-shattering. On the other hand, OG-B and OG-C1 were the only groups that were fixed for the *sh4* casual domestication mutation while the *sh1* gene was wildtype, and many individuals had higher proportions of shattering seeds. This indicates mutations in at least two shattering genes (in the case of the OG-A2, OG-B, and OG-C group the genes *sh1 and sh4*) were required for complete non-shattering in *O*. *glaberrima*. In the case of OG-B and OG-C1 group, selection for non-shattering was incomplete either because the group represents an ancestral population or is a result from the cultural preference on the degree of seed shattering.

Samples closer to the coast and belonging to the groups OG-A1, OG-A2, and OG-C2 were predominantly non-shattering rice. Interestingly for the OG-A1 group, the casual domestication mutation was polymorphic in both *sh1* and *sh4* genes (Table 1) but all varieties in OG-A1 had non-shattering seeds (Fig 8B). There were 27 OG-A1 individuals with the same *sh1* and *sh4* allelic status (i.e. *sh1* wildtype and *sh4* mutant) as the OG-B and OG-C1 group (S12 Table). However, unlike the inland group, all individuals had non-shattering seeds suggesting there may be a third shattering gene, and/or different mutations in *sh1* involved in the non-shattering phenotype. In addition, all seed non-shattering OG-A1 individuals without the casual *sh4* mutation (Fig 6B star) had the *sh1* deletion (S12 Table). This suggests the casual mutations for *sh1* and *sh4* were independently selected, possibly from different genetic backgrounds.

In the end, our panicle threshability results are consistent with the population genetics result of *sh1* and *sh4*. Specifically, the selection process for non-shattering was either incomplete (i.e. OG-B and OG-C1 population) or heterogeneous (i.e. OG-A and OG-C2 population), where two individuals with the same degree of threshability did not share the same casual domestication mutations in their shattering genes (i.e. OG-A1 population). This opens up the possibility that domestication, at least involving seed non-shattering, does not have a single origin in *O*. *glaberrima*, but may have occurred in multiple genetic backgrounds and/or geographical regions.

### Conclusion

Our analysis of whole genome re-sequencing data in the African rice *O*. *glaberrima* and its wild ancestor *O*. *barthii* provides key insights into the geographic structure and nature of domestication in crop species. Our analysis suggests that *O*. *glaberrima* is comprised of at least 5 distinct genetic groups, which are found in different geographic areas in West and Central Africa. We find that many individuals that have been identified as *O*. *barthii* (and which in the past have been thought to be the immediate ancestor of the domesticated crop) form a distinct genetic group that behaves almost identically to *O*. *glaberrima*. These include similarities in LD decay, polymorphism levels, and low genetic differentiation with domesticated African rice. Moreover, several of these *O*. *barthii* individuals carried causal mutations in the key domestication genes *sh4*, *sh1* and *PROG1*. Together this suggests these *O*. *barthii* individuals, which we collectively refer to as the OB-G group, may represent a feral *O*. *glaberrima* or may have been misidentified as the crop species.

Portères hypothesized that western inland Africa near the inner Niger delta of Mali as the center of origin for *O*. *glaberrima* [8,9], and this has been the commonly accepted domestication model for *O*. *glaberrima* [84]. Under this single center of origin model, *O*. *glaberrima* from the OG-C1 genetic group (closest to the inner Niger delta) would have acquired key domestication mutations before spreading throughout West Africa. Here, we suggest that the domestication of *O*. *glaberrima* may be more complex. Phylogeographic analysis of three domestication loci indicates that the causal mutations associated with the origin of *O*. *glaberrima* may have arisen in three different areas. Phenotype assay of panicle threshability, a core early plant domestication trait [28], showed that the selection for seed non-shattering was incomplete in several inland *O*. *glaberrima* samples. Within the coastal *O*. *glaberrima* samples, almost all individuals have non-shattering seeds but the casual domestication mutations in two key shattering genes (*sh1* and *sh4*) are not fixed.

Our results support a view, in which domestication has largely been a long protracted process, often involving thousands of years of transitioning a wild plants into a domesticated state [82,85,86]. If this indeed happened for *O*. *glaberrima*, our study suggests this protracted period of domestication had no clear single center of domestication in African rice. Instead domestication of African rice was likely a diffuse process involving multiple centers [86–88]. In this model, cultivation may have started at a location and proto-domesticates spread across the region with some (but maybe not all) domestication alleles. Across the multiple regions, the differing environmental conditions and cultural preferences of the people domesticating this proto-*glaberrima* resulted in differentiation into distinct genetic groups. Temporal and spatial variation in the domestication genes resulted in causal mutations for domestication traits appearing at different parts of the species range. The genetic and geographic structure in this domesticated species suggests that admixture might have allowed local domestication alleles to spread into other proto-domesticated *O*. *glaberrima* genetic groups in different parts of West and Central Africa. This would have facilitated the development of modern domesticated crop species, which contain multiple domestication alleles sourced from different areas. In the end, these gradual changes occurring across multiple regions provided different mutations at key domestication genes, which ultimately spread and came together to form modern *O*. *glaberrima*.

There has been intense debate on the nature of domestication, and recently (with particular emphasis on early Fertile Crescent domestication) discussion on whether this process proceeds in localized (centric) vs. a diffuse manner across a wider geographic area (non-centric) [82,87,89]. As we begin to use more population genomic data and whole genome sequences, as well as identify causal mutations associated with key domestication traits, we can begin to study the interplay between geography, population structure and the evolutionary history of specific domestication genes and reconstruct the evolutionary processes that led to the origin and domestication of crop species. Moreover, a functional phylogeographic approach, as demonstrated here, could provide geographic insights into key traits that underlie species characteristics, and may allow us to understand how functional traits originate and spread across a landscape.

## Materials and methods

### Sample genome sequencing

*O*. *glaberrima* and *O*. *barthii* samples were ordered from the International Rice Research Institute and their accession numbers can be found in S1 Table. DNA was extracted from a seedling stage leaf using the Qiagen DNeasy Plant Mini Kit. Extracted DNA from each sample was prepared for Illumina genome sequencing using the Illumina Nextera DNA Library Preparation Kit. Sequencing was done on the Illumina HiSeq 2500 –HighOutput Mode v3 with 2×100 bp read configuration, at the New York University Genomics Core Facility. Raw FASTQ reads are available from NCBI biproject ID PRJNA453903.

### Reference genome based read alignment

Raw FASTQ reads from the study Wang et al. [13] and Meyer et al. [14] were downloaded from the sequence read archive (SRA) website with identifiers SRP037996 and SRP071857 respectively.

FASTQ reads were preprocessed using BBTools (https://jgi.doe.gov/data-and-tools/bbtools/) bbduk program version 37.66 for read quality control and adapter trimming. For bbduk we used the option: minlen = 25 qtrim = rl trimq = 10 ktrim = r k = 25 mink = 11 hdist = 1 tpe tbo; which trimmed reads below a phred score of 10 on both sides of the reads to a minimum length of 25 bps, 3' adapter trimming using a kmer size 25 and using a kmer size of 11 for ends of read, allowing one hamming distance mismatch, trim adapters based on overlapping regions of the paired end reads, and trim reads to equal lengths if one of them was adapter trimmed.

FASTQ reads were aligned to the reference *O*. *glaberrima* genome downloaded from EnsemblPlants release 36 (ftp://ftp.ensemblgenomes.org/pub/plants/). Read alignment was done using the program bwa-mem version 0.7.16a-r1181 [90]. Only the 12 pseudomolecules were used as the reference genome and the unassembled scaffolds were not used. PCR duplicates during the library preparation step were determined computationally and removed using the program picard version 2.9.0 (http://broadinstitute.github.io/picard/).

### Sequence alignment analysis

Using the BAM files generated from the previous step, genome-wide read depth for each sample was determined using GATK version 3.8–0 (https://software.broadinstitute.org/gatk/).

Because of the differing genome coverage between samples generated from different studies, depending on the population genetic method we used different approaches to analyze the polymorphic sites. A complete probabilistic framework without hard-calling genotypes, was implemented to analyze levels of polymorphism (including estimating θ, Tajima’s D, and F_ST_), population relationships (ancestry proportion estimation and phylogenetic relationship), and admixture testing (ABBA-BABA test). For methods that require genotype calls, we analyzed samples that had greater then 10× genome coverage. Details are shown below.

### Polymorphism analysis

We used ANGSD version 0.913 [53] and ngsTools [54] which uses genotype likelihoods to analyze the polymorphic sites in a probabilistic framework. ngsTools uses the site frequency spectrum as a prior to calculate allele frequencies per site. To polarize the variants the *O*. *rufipogon* genome sequence [36] was used. Using the *O*. *glaberrima* genome as the reference, the *O*. *rufipogon* genome was aligned using a procedure detailed in Choi et al. [37]. For every *O*. *glaberrima* genome sequence position, the aligned *O*. *rufipogon* genome sequence was checked, and changed to the *O*. *rufipogon* sequence to create an *O*. *rufipogon*-ized *O*. *glaberrima* genome. Gaps, missing sequence, and repetitive DNA were noted as ‘N’. For all analysis we required the minimum base and mapping quality score per site to be 30. We excluded repetitive regions in the reference genome from being analyzed, as read mapping to these regions can be ambiguous and leading to false genotypes.

The site frequency spectrum was then estimated using ANGSD with the command:

angsd–b $BAM_list -out $SFS \
  –ref $Reference_Genome–anc $Outgroup_genome \
  -uniqueOnly 1 –remove_bads 1 –only_proper_pairs 1 \
  –trim 0 –C 50 –baq 1 –minMapQ 30 –minQ 30 -minInd $minInd \
  –setMinDepth $setMinDepth–setMaxDepth $setMaxDepth \
  -doCounts 1 –GL 1 –doSaf 1

For each genetic group a separate site frequency spectrum was estimated and the options–minInd, -setMinDepth, and–setMaxDepth were changed accordingly. Parameter minInd represent the minimum number of individuals per site to be analyzed, setMinDepth represent minimum total sequencing depth per site to be analyzed, and setMaxDepth represent maximum total sequencing depth per site to be analyzed. We required–minInd to be 80% of the sample size, -setMinDepth to be one-third the average genome-wide coverage, and–setMaxDepth to be 2.5 times the average genome-wide coverage. Using the site frequency spectrum, θ was calculated with the command:

angsd–b $BAM_list -out $Theta \
  –ref $Reference_Genome–anc $Outgroup_genome \
  -uniqueOnly 1 –remove_bads 1 –only_proper_pairs 1 \
  –trim 0 –C 50 –baq 1 –minMapQ 30 –minQ 30 -minInd $minInd \
  –setMinDepth $setMinDepth–setMaxDepth $setMaxDepth \
  -doCounts 1 –GL 1 –doSaf 1 –doThetas 1 –pest $SFS

The θ estimates from the previous command was used to compute sliding window values for Tajima’s θ and D [71] with the command:

thetaStat do_stat $Theta–nChr $Indv–win 10000 –step 10000

The option nChr is used for the total number of samples in the group being analyzed. Window size was set as 10,000 bp and was incremented in non-overlapping 10,000 bp.

F_ST_ values between pairs of population were also calculated using a probabilistic framework. Initially, we calculated the joint site frequency spectrum (2D-SFS) between the two populations of interest with the command:

realSFS $Pop1_SFS $Pop2_SFS > $Pop1_Pop2_2DSFS

Each population’s site frequency spectrum estimated from previous step is used to estimate the 2D-SFS. With the 2D-SFS F_ST_ values were calculated with the command:

realSFS fst index $Pop1_SFS $Pop2_SFS \
  –sfs $Pop1_Pop2_2DSFS–fstout $Pop1_Pop2_Fst

F_ST_ values were calculated in non-overlapping 10,000 bp sliding windows. For the sliding windows calculated for θ, Tajima’s D, and F_ST_ values, we required each window to have at least 30% of the sites with data or else the window was discarded from being analyzed.

### Determining population relationships

Ancestry proportions were estimated using NGSadmix [55]. Initially, genotype likelihoods were calculated using ANGSD with the command:

angsd–b $BAM_list -out $GL \
  –ref $Reference_Genome–anc $Outgroup_genome \
  -uniqueOnly 1 –remove_bads 1 –only_proper_pairs 1 \
  –trim 0 –C 50 –baq 1 –minMapQ 30 –minQ 30 -minInd $minInd \
  –setMinDepth $setMinDepth–setMaxDepth $setMaxDepth \
  -doCounts 1 –GL 1 –doMajorMinor 4 –doMaf 1 \
  -skipTrialleic 1 –doGlf 2 –SNP_pval 1e-3

To reduce the impact of LD would have on the ancestry proportion estimation, we randomly picked a polymorphic site in non-overlapping 50 kbp windows. In addition we made sure that the distance between polymorphic sites were at least 25 kbp apart. We then used NGSadmix to estimate the ancestry proportions for K = 2 to 9. For each K the analysis was repeated 100 times and the ancestry proportion with the highest log-likelihood was selected to represent that K.

Phylogenetic relationships between samples were reconstructed using the genetic distance between individuals. Distances were estimated using genotype posterior probabilities from ANGSD command:

angsd–b $BAM_list -out $GPP \
  –ref $Reference_Genome–anc $Outgroup_genome \
  -uniqueOnly 1 –remove_bads 1 –only_proper_pairs 1 \
  –trim 0 –C 50 –baq 1 –minMapQ 30 –minQ 30 -minInd $minInd \
  –setMinDepth $setMinDepth–setMaxDepth $setMaxDepth \
  -doCounts 1 –GL 1 –doMajorMinor 4 –doMaf 1 \
  -SNP_pval 1e-3 –doGeno 8 –doPost 1

Genotype posterior probability was used by NGSdist [58] to estimate genetic distances between individuals, which was then used by FastME ver. 2.1.5 [91] to reconstruct a neighbor-joining tree. Tree was visualized using the website iTOL ver. 3.4.3 (http://itol.embl.de/) [92].

Principal component analysis were also conducted using genotype likelihoods. Genotype posterior probabilities from ANGSD command:

angsd–b $BAM_list -out $GPP \
  –ref $Reference_Genome–anc $Outgroup_genome \
  -uniqueOnly 1 –remove_bads 1 –only_proper_pairs 1 \
  –trim 0 –C 50 –baq 1 –minMapQ 30 –minQ 30 -minInd $minInd \
  –setMinDepth $setMinDepth–setMaxDepth $setMaxDepth \
  -doCounts 1 –GL 1 –doMajorMinor 1 –doMaf 1 –skipTriallelic 1 \
  -SNP_pval 1e-3 –doGeno 32 –doPost 1

The genotype posterior probability was then used by the program ngsCovar [54] to conduct the principal component analysis.

### SNP calling

Since several methods require genotype calls for analysis SNP calling was also performed. Samples with greater than or equal to 10× genome coverage (GE10 dataset) was considered to ensure sufficient read coverage for each site at the cost of excluding individuals from genotype calling. These were 174 individuals that belonged to the genetic grouping designated by this study, and full list of individuals can be found in S13 Table.

For each sample, genotype calls for each site was conducted using the GATK HaplotypeCaller engine under the option `-ERC GVCF`mode to output as the genomic variant call format (gVCF). The gVCFs from each sample were merged together to conduct a multi-sample joint genotyping using the GATK GenotypeGVCFs engine. Genotypes were divided into SNP or INDEL variants and filtered using the GATK bestpractice hard filter pipeline [93]. For SNP variants we excluded regions that overlapped repetitive regions and variants that were within 5 bps of an INDEL variant. We then used vcftools version 0.1.15 [94] to select SNPs that had at least 80% of the sites with a genotype call, and exclude SNPs with minor allele frequency <2% to remove potential false positive SNP calls arising from sequencing errors or false genotype calls.

### Preparing data for determining *O*. *glaberrima* population relationships

The GE10 dataset was used for this analysis, as it requires hard-called genotypes. The *O*. *glaberrima* samples were grouped according to the grouping scheme designated in this study (Fig 3), and any members that were more similar to other grouping then its own were examined by estimating their silhouette scores [65]. Using the program PLINK version 1.9 [95] for calculating genetic distances from all pairwise comparisons, silhouette scores were calculated using the formula:

s(i)=[b(i)−a(i)]/max{a(i),b(i)} (1)

where *i* represents an individual, *s* the silhouette score, *a* the average genetic distance to members of own group, and *b* the average genetic distance to members of foreign group. Individuals with negative silhouette scores were filtered out. After filtering, using the remaining individuals the silhouette score based filtering method was iteratively conducted until all individuals had silhouette scores higher then 0.1.

To obtain the outgroup nucleotide variants we downloaded raw sequencing data for six *O*. *rufipogon* species corresponding to the Or-C and Or-D clade, which were shown to contain the least amount of domesticated Asian rice admixture from feralization [96]. These samples have identifiers W0137, W1739, W1807, W0170, W0630, and W2263 with SRA run accession IDs of DRR088674, ERR224552, DRR088680, ERR2245549, DRR001185, and DRR088691. *O*. *rufipogon* raw FASTQ reads were aligned to the *O*. *glaberrima* reference genome as outlined in our previous steps. GATK HaplotypeCaller engine was used for calling genotypes but the multi-sample joint genotyping step for the six *O*. *rufipogon* samples were limited to polymorphic sites that overlapped the SNP positions analyzed in the silhouette score analysis.

### Treemix analysis

Population relationships were examined as admixture graphs using Treemix version 1.13 [67]. SNP calls from the core set population was used to calculate the allele frequencies for each genetic group. One hundred SNPs were analyzed together as a block to account for the effects of LD between SNPs. The *O*. *rufipogon* variation was used as the outgroup and a Treemix model assuming 0–3 migration events were fitted. The four-population test [68] was conducted using the fourpop program from the Treemix package.

### Estimating levels of linkage disequilibrium

Genome-wide levels of LD (r^2^) was estimated with the GE10 dataset and using the program PLINK. LD was calculated for each genetic group separately across a non-overlapping 1Mbp window and between variants that are at most 99,999 SNPs apart. LD data was summarized by calculating the mean LD between a pair of SNPs in 1,000 bp bins. A LOESS curve fitting was applied for a line of best fit and to visualize the LD decay.

### Determining gene orthologs between Asian and African Rice

We downloaded protein coding sequences for *O*. *sativa*, *O*. *glaberrima*, and *O*. *barthii* from EnsemblPlants release 36. An all-vs-all reciprocal BLAST hit approach was used to determine orthologs between species and paralogs within species. We used the program Orthofinder ver. 1.19 [97] to compare the proteomes between and within species for ortholog assignment. Orthofinder used the program DIAMOND ver. 0.8.37 [98] for sequence comparisons.

### Gene deletion analysis of genes *sh1* and *PROG1*

Synteny based on the *O*. *sativa sh1* gene (*Ossh1*; *O*. *sativa cv*. *japonica* chromosome 3:25197057–25206948) indicated orthologs surrounding *Ossh1* was found in chromosome 3 of *O*. *barthii* and on an unassembled scaffold named Oglab03_unplaced035 in *O*. *glaberrima* (S14 Table). The *sh1* gene was missing in *O*. *glaberrima* suggesting the gene deletion may have led to complex rearrangements that prevented correct assembly of the region in the final genome assembly. Because of this we used the *O*. *barthii* genome sequence to align raw reads and call polymorphic sites for downstream analysis.

The approximate region of the deletion in the *O*. *barthii* genome coordinate was examined by looking at the polymorphic sites, since our quality control filter removed polymorphic sites if it had less than 80% of the individuals with a genotype call. Between the genomic positions at *O*. *barthii* chromosome 3 position 23,100,000–23,130,000, no polymorphic sites passed the quality control filter (S13 Fig) and contained the gene *Obsh1*. Between the region at *O*. *barthii* chromosome 7 position 2,655,000–2,675,000 there was also no polymorphisms passing the filter and contained the gene *ObPROG1* (S14 Fig).

Gene deletion was inferred from comparing the read depth of a genic region inside and outside a candidate deletion region. Read depth was measured using bedtools ver. 2.25.0 [99] genomecov program. Individuals with and without the deletion were determined by comparing the median read coverage of the domestication gene within the candidate deletion region, to a gene that is outside the deletion region. We checked the orthologs to make sure the gene outside the deletion region existed in *O*. *barthii*, *O*. *glaberrima*, and *O*. *sativa*. To determine the *sh1* deletion status we examined its read depth and compared it to the *O*. *barthii* gene OBART03G27620 that was upstream and outside the candidate deletion region. Ortholog of OBART03G27620 is found in both *O*. *sativa* (Os03g0648500) and *O*. *glaberrima* (ORGLA03G0257300). To determine the deletion status of *PROG1* gene we examined its read depth and compared to *O*. *barthii* gene OBART07G03440. Ortholog of OBART07G03440 is found in both *O*. *sativa* (Os07g0153400) and *O*. *glaberrima* (ORGLA07G0029300).

Because some individuals had low genome-wide coverage (S1 Table) there is the possibility that some of those individuals had been detected as false positive deletion events. There are two main reasons we believe the deletions are likely to be present even for low coverage individuals. For example for the *sh1* deletion, (i) all individuals had at least a median coverage of ~1× in the OBART03G27620 gene (S15 Table) suggesting read coverage may be low but if the gene is not deleted it is evenly distributed across a gene, and (ii) even comparing individuals with and without the *sh1* deletion that had a ~1× median coverage in the non-deleted OBART03G27620 gene, there were clear differences in the *sh1* gene coverage (S15 Fig) where the individuals with the deletion always had a median coverage of zero.

### Shattering gene nomenclature

Gene names for the non-shattering phenotype have unfortunately varied between different *Oryza* studies. Genetic studies comparing Asian rice *O*. *sativa cv*. Japonica and its wild progenitor *O*. *rufipogon* had identified a single dominant allele responsible for non-shattering and named the locus as *Sh3* [100,101]. The causal gene was later identified on chromosome 4 and was given a new name as *sh4* [17]. Studies have used the names *Sh3* and *sh4* synonymously as the common gene name for the gene with locus ID Os04g0670900 [78].

Lv et al. [81] had found an *O*. *glaberrima* specific gene deletion in chromosome 3 that caused a non-shattering phenotype and named this gene as *SH3*. *SH3* belongs to a YABBY protein family transcription factor. Using the *SH3* coding sequence in *O*. *barthii* (*ObSH3*), which the gene is not deleted, orthologs were found in maize (B4FY22), barley (M0YM09), and Brachypodium (I1GPY5) [81]. We discovered this group of proteins belonged to a group identified in Plant Transcription Factor Database ver 4.0 [102] under the ID OGMP1394. The *O*. *sativa* gene member of this group was gene ID Os03g0650000, which has previously been identified as a gene involved in non-shattering [103]. Thus, *ObSH3* and Os03g0650000 are orthologs of each other and Os03g0650000 has been named as *sh1*. Here, we followed the guideline recommended by Committee on Gene Symbolization Nomenclature and Linkage (CGSNL) [104] to designate *SH3* from Lv et al. [81] as *sh1* to avoid using the overlapping gene name *sh3*.

### Gene haplotype analysis

To investigate the haplotype structure around the domestication genes we used all individuals from *O*. *glaberrima*, OB-G, and OB-W population regardless of the genome coverage. The *O*. *glaberrima* and *O*. *barthii* genome were used as reference to align the raw reads and call polymorphisms as outlined above. Missing genotypes were then imputed and phased using Beagle version 4.1 [105].

We used vcftools to extract polymorphic sites around a region of interest. The region was checked for evidence of recombination using a four-gamete test [80], to limit the edges connecting haplotypes as mutation distances during the haplotype network reconstruction. To minimize false positive four-gamete test results caused from technical errors such as genotype error and sequencing error, if the observed frequency of the fourth haplotype was below 1% we considered the haplotype an error and did not consider it as evidence of recombination. If a region had evidence of recombination we checked if the recombination was limited to the wild or domesticated African rice. If recombination was only detected in the wild population then we determined the pair of SNPs that failed the four-gamete test. Here, because the four-gamete test did not detect any evidence of recombination in the *O*. *glaberrima* population, the fourth haplotype observed in the wild population is only limited to *O*. *barthii* and do not provide any information with regard to the direct origin of the *O*. *glaberrima* haplotypes. Hence, we removed individuals with the fourth haplotype and estimated the haplotype network of the region.

Haplotype network was reconstructed using the R pegas [106] and VcfR [107] package, using the hamming distance between haplotypes to construct a minimum spanning tree. For each domestication gene and its surrounding region, a phylogenetic tree was reconstructed by sampling a single haplotype for each individual. Bootstrap replicated phylogenetic trees were built using RAxML [108] and plotted with iTOL.

### Seed threshability measurement

*O*. *glaberrima* landraces were grown during the 2018 dry season at the International Rice Research Institute (IRRI) block L4 (14°09'34.6"N 121°15'42.4"E) experimental field. At maturity, when at least 85% of the grains on a panicle are matured [109,110], panicles were harvested and evaluated for threshability using a established method by IRRI [111]. In brief, a total of 6 plants for each landrace from three plot replicates were sampled. During panicle threshability measurement, each panicle was grasped to apply slight pressure. Grains detached from the panicle and panicles intact with grains were collected. The numbers of grains that detached and remained attached were counted separately to obtain the percentage of shattered grains [83]. Percent shattering were converted to panicle threshability scores according to the Standard Evaluation System for Rice [83].

## Supporting information

S1 Fig Geographic distribution of analyzed *O*. *glaberrima* and *O*. *barthii* samples. (A) *O*. *glaberrima* from this study. (B) *O*. *glaberrima* from Meyer et al. (C) *O*. *barthii* samples. Note for the majority of *O*. *barthii* samples from Wang et al. the locations are unknown.
(TIF)

S2 Fig Ancestry proportion estimates for K = 2 to 9. Black stars below the admixture barplot indicate *O*. *glaberrima* individuals. Colored stars above admixture barplot are the *O*. *barthii* grouping designated by Wang et al. where blue: OB-I, brown: OB-II, red: OB-III, yellow: OB-IV, and pink: OB-V group.
(TIF)

S3 Fig Neighbor-joining tree built using a distance matrix estimated from NGSdist. Color strips represent the *O*. *barthii* grouping designated by Wang et al.
(TIF)

S4 Fig Ancestry proportion estimates for K = 2, 5, and 7. Black stars below the admixture barplot indicate the two *O*. *glaberrima* individuals IRGC103631 and IRGC103638. Colored stars above admixture barplot are the *O*. *barthii* grouping designated by Wang et al. where blue: OB-I, brown: OB-II, red: OB-III, yellow: OB-IV, and pink: OB-V group.
(TIF)

S5 Fig MDS plot and silhouette scores for individuals before (A,B) and after (C,D) the silhouette score based filtering step. (A,C) MDS plot of genetic variation. (B,D) Genetic distance based silhouette scores.
(TIF)

S6 Fig MDS plot of the *O*. *glaberrima* core set population, and their outgroups. (TIF)

S7 Fig Treemix results and residual plot for model assuming 3 migration events. (TIF)

S8 Fig Levels of polymorphism and Tajima’s D for OB-G, OB-W and *O*. *glaberrima*. Significant difference after Mann-Whitney U test (p < 0.001) are indicated with three stars.
(TIF)

S9 Fig π_w_/ π_D_ statistics around the *PROG1* region in *O*. *barthii* and *O*. *glaberrima* reference genomes. (TIF)

S10 Fig Maximum-likelihood tree of up and downstream 25 kbp or 50 kbp of the 3 domestication genes. Light grey represent O. barthii while dark grey represent O. glaberrima individuals. (A) Tree for PROG1 region. Black arrows indicate the two wild rice that are sister to all O. glaberrima samples. (B) Tree for sh4 region. Star indicates the individuals without the nonsense mutation. (C) Tree for sh1 region. Nodes with greater then 90% bootstrap support are shown with circles.
(TIF)

S11 Fig Haplotype network of the downstream 5 kbp of the PROG1 deletion.
(TIF)

S12 Fig πw/ πD statistics around the sh1 region in O. barthii reference genome for O. glaberrima individuals with (left) and without (right) the sh1 deletion.
(TIF)

S13 Fig Genome coordinate of chromosome 3 and presence of a polymorphism is indicated with a point. (TIF)

S14 Fig Genome coordinate of chromosome 7 and presence of a polymorphism is indicated with a point. (TIF)

S15 Fig Visualization of read pileup of a region upstream and a region within the *Obsh1* gene for individuals with and without the *sh1* deletion. In each panel an individual with low and high coverage are compared.
(TIF)

S1 Table Information on the sequenced and analyzed individuals of this study. (XLSX)

S2 Table Count of country of origin for individuals from OB-G and OB-W genetic group. (XLSX)

S3 Table *O*. *barthii* and *O*. *glaberrima* individuals consisting of the core set population. (XLSX)

S4 Table *f4* test results testing tree-ness involving the *O*. *glaberrima* groups OG-A and OG-B. (XLSX)

S5 Table *O*. *glaberrima* and *O*. *barthii* genes syntenic to the *O*. *sativa PROG1* region. (XLSX)

S6 Table Individuals corresponding to the haplotype groups identified in Fig 4A *PROG1* upstream gene region. (XLSX)

S7 Table Individuals corresponding to the haplotype groups identified in S11 Fig *PROG1* downstream gene region. (XLSX)

S8 Table Individuals corresponding to the haplotype groups identified in Fig 6B *sh4* gene region. (XLSX)

S9 Table Individuals corresponding to the haplotype groups identified in Fig 6C *sh1* gene region. (XLSX)

S10 Table *O*. *glaberrima* samples and their shattering percentage and scores. (XLSX)

S11 Table Bonferroni-corrected Mann-Whitney U test p-values comparing shattering scores between genetic groups. (XLSX)

S12 Table OG-A1 group samples *sh4* and *sh1* haplogroup and mutations status, and their shattering scores. (XLSX)

S13 Table Individuals with greater then 10× genome coverage. (XLSX)

S14 Table *O*. *glaberrima* and *O*. *barthii* genes syntenic to the *O*. *sativa sh1* region. (XLSX)

S15 Table Read coverage count for *sh1* and *PROG1* region. (XLSX)
