# Supplementary figures and images for: The complex geography of domestication of the African rice Oryza glaberrima
Source: PLoS Genet. 2019 Mar 7;15(3):e1007414. doi: 10.1371/journal.pgen.1007414 (PMC6424484; doi:10.1371/journal.pgen.1007414)

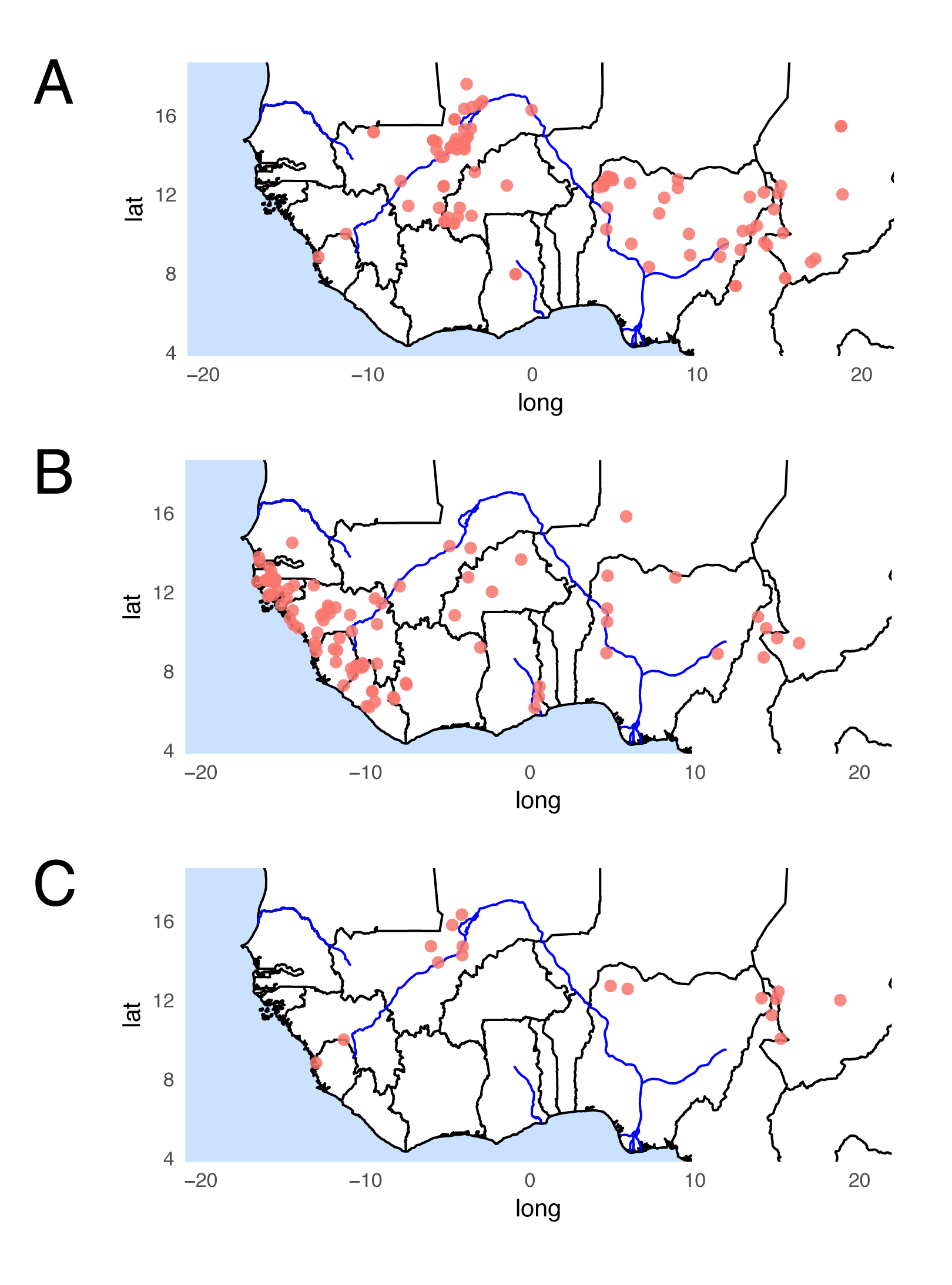

Supplement: S1 Fig — (A) O. glaberrima from this study. (B) O. glaberrima from Meyer et al. (C) O. barthii samples. Note for the majority of O. barthii samples from Wang et al. the locations are unknown. (TIF) [file pgen.1007414.s001.tif]

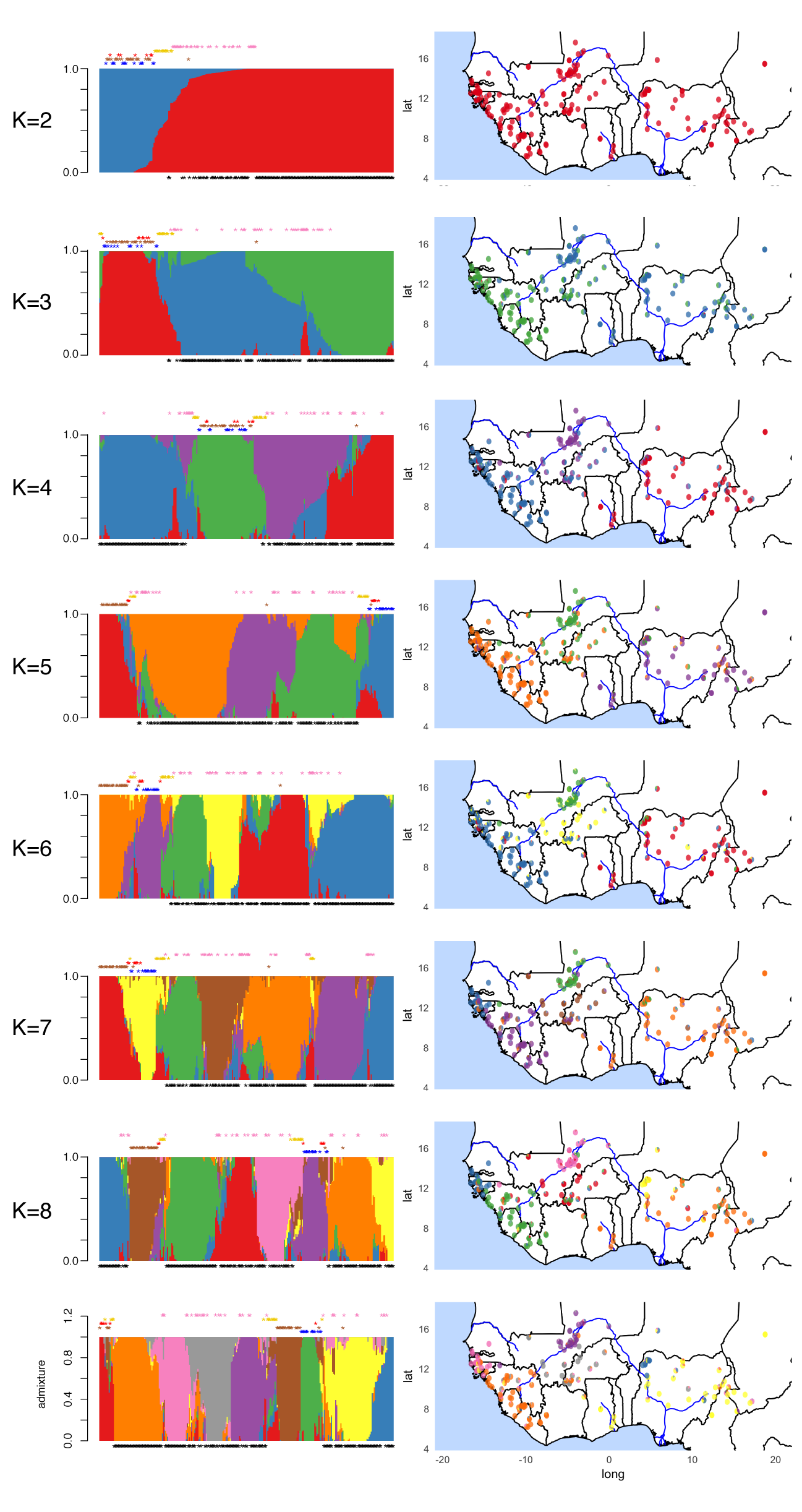

Supplement: S2 Fig — Black stars below the admixture barplot indicate O. glaberrima individuals. Colored stars above admixture barplot are the O. barthii grouping designated by Wang et al. where blue: OB-I, brown: OB-II, red: OB-III, yellow: OB-IV, and pink: OB-V group. (TIF) [file pgen.1007414.s002.tif]

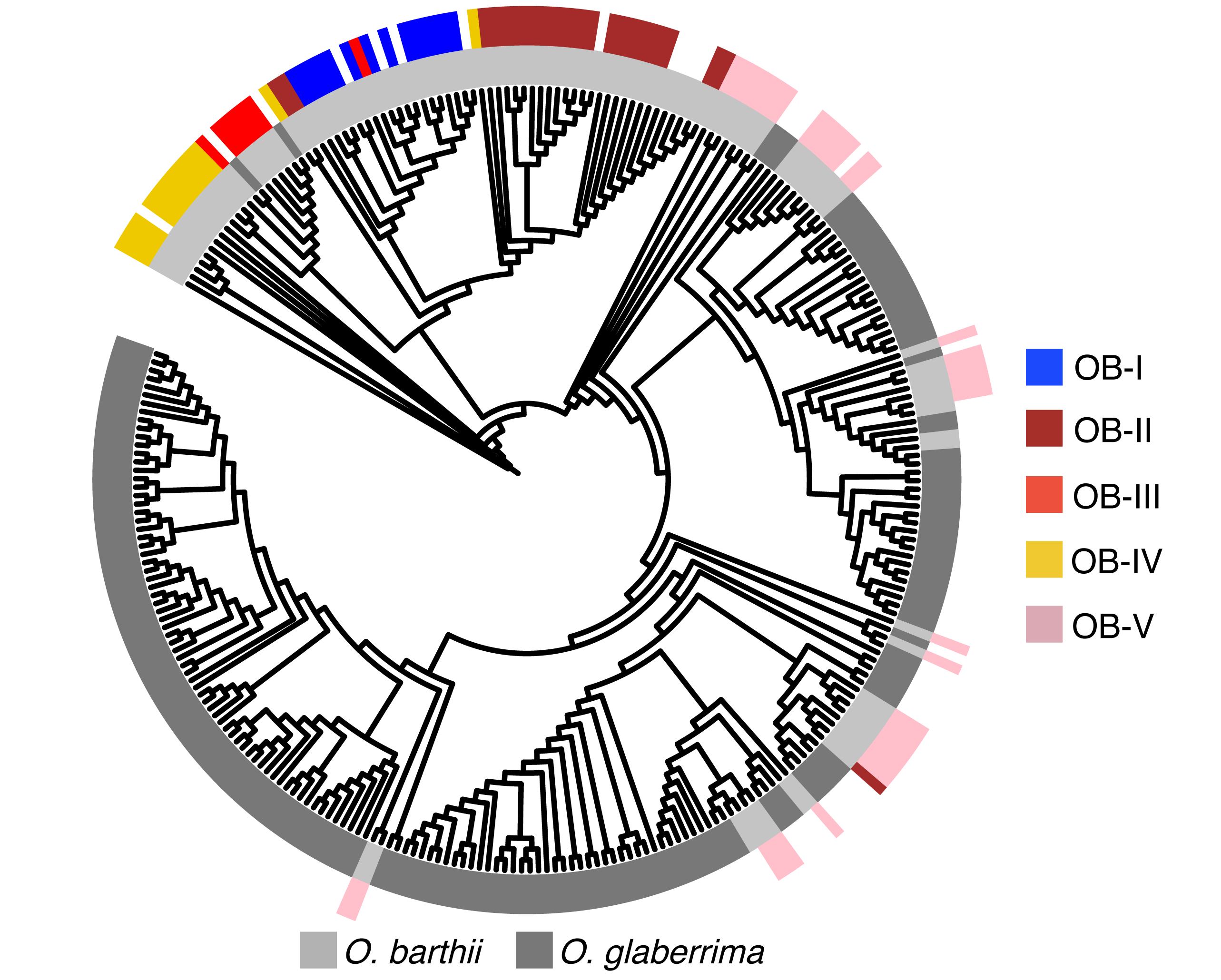

Supplement: S3 Fig — Color strips represent the O. barthii grouping designated by Wang et al. (TIF) [file pgen.1007414.s003.tif]

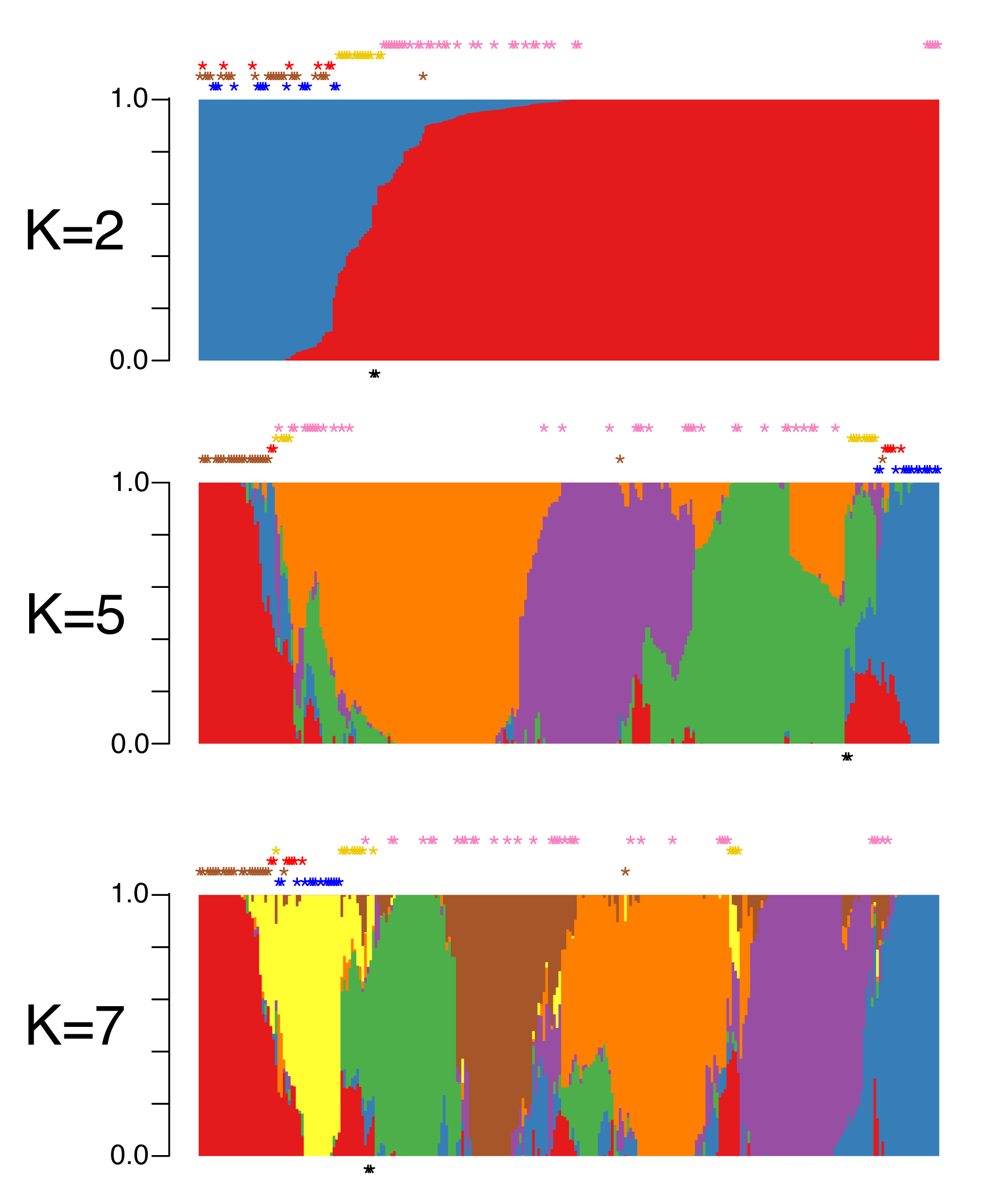

Supplement: S4 Fig — Black stars below the admixture barplot indicate the two O. glaberrima individuals IRGC103631 and IRGC103638. Colored stars above admixture barplot are the O. barthii grouping designated by Wang et al. where blue: OB-I, brown: OB-II, red: OB-III, yellow: OB-IV, and pink: OB-V group. (TIF) [file pgen.1007414.s004.tif]

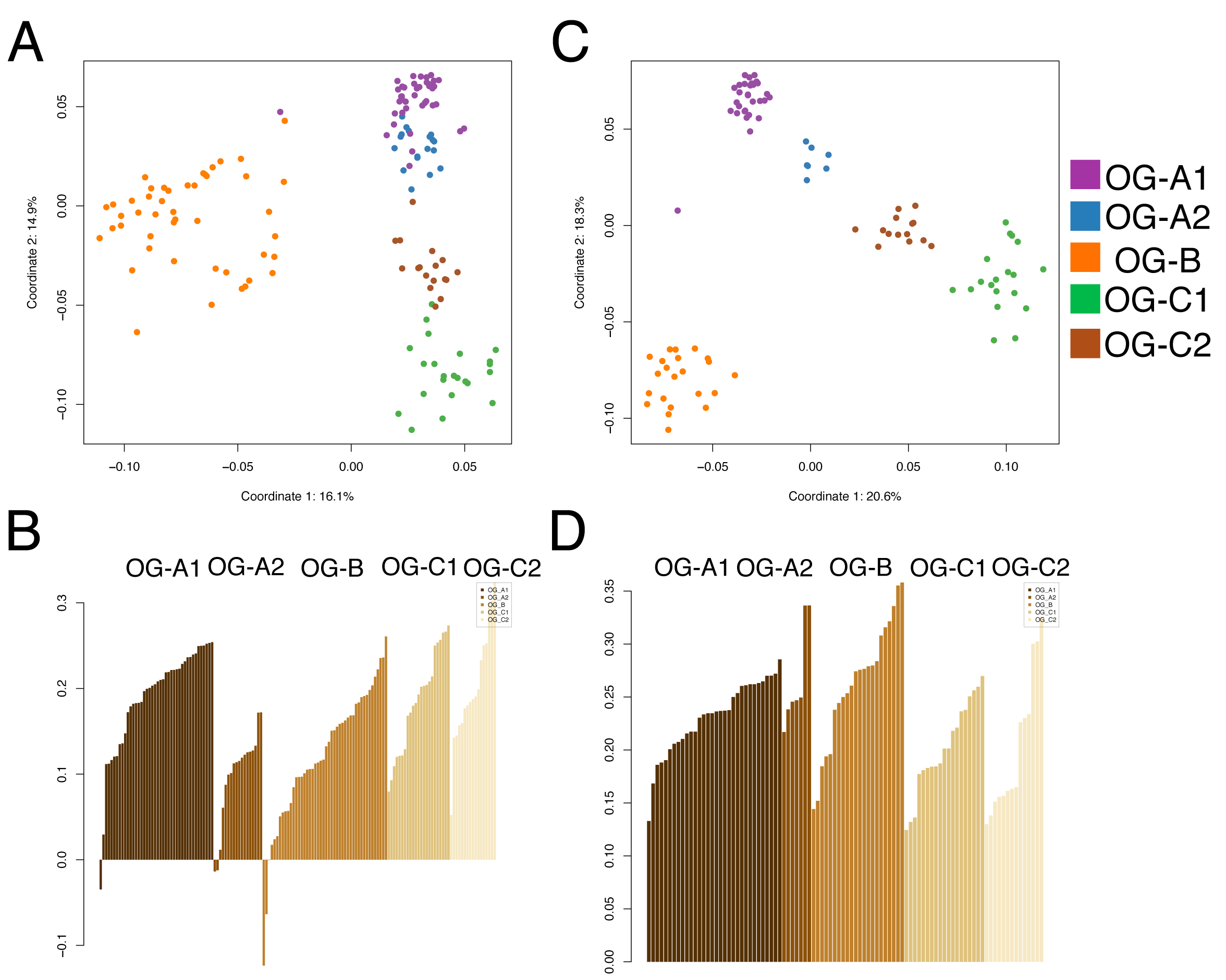

Supplement: S5 Fig — MDS plot and silhouette scores for individuals before (A,B) and after (C,D) the silhouette score based filtering step. (A,C) MDS plot of genetic variation. (B,D) Genetic distance based silhouette scores. (TIF) [file pgen.1007414.s005.tif]

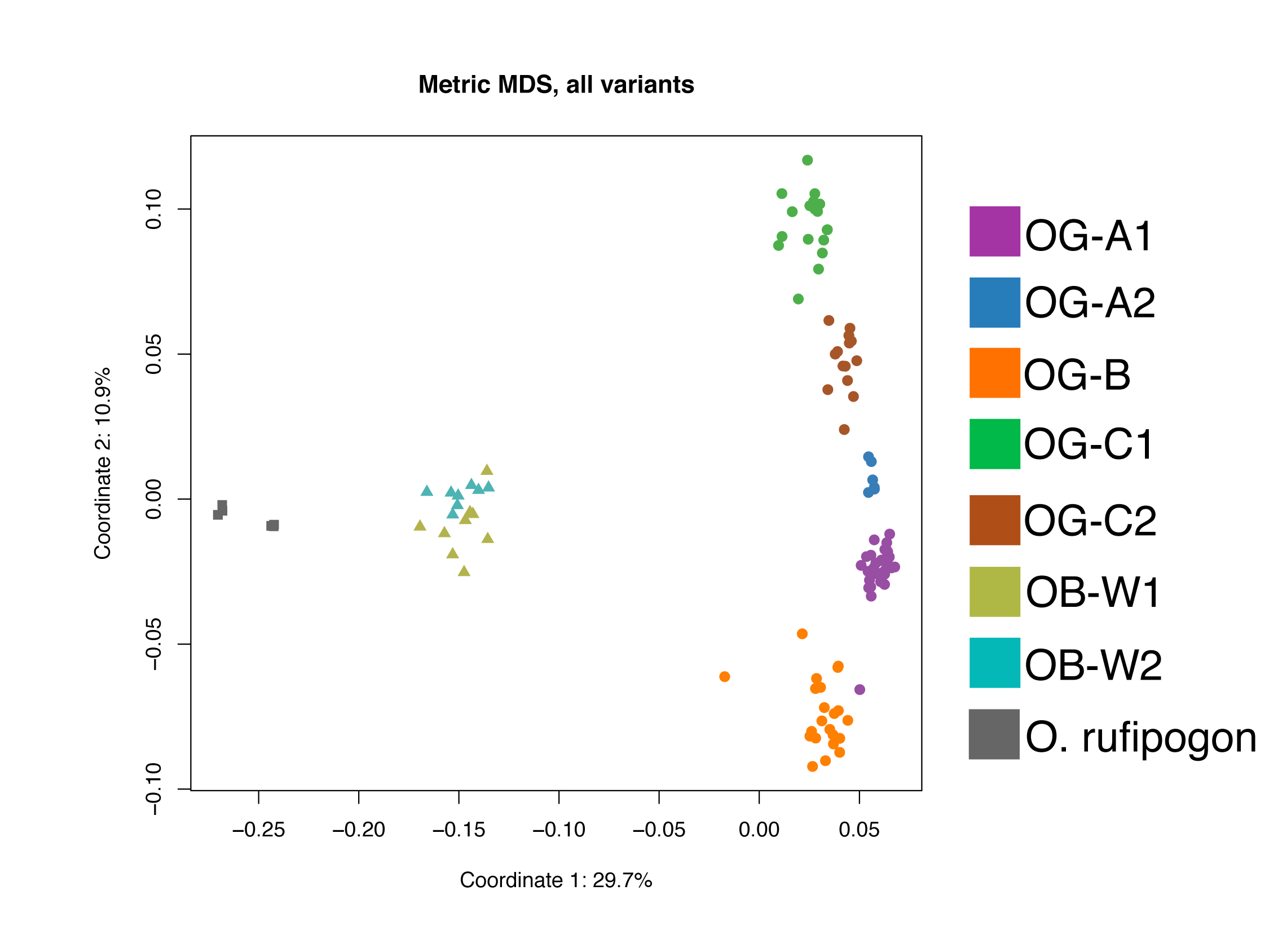

Supplement: S6 Fig — (TIF) [file pgen.1007414.s006.tif]

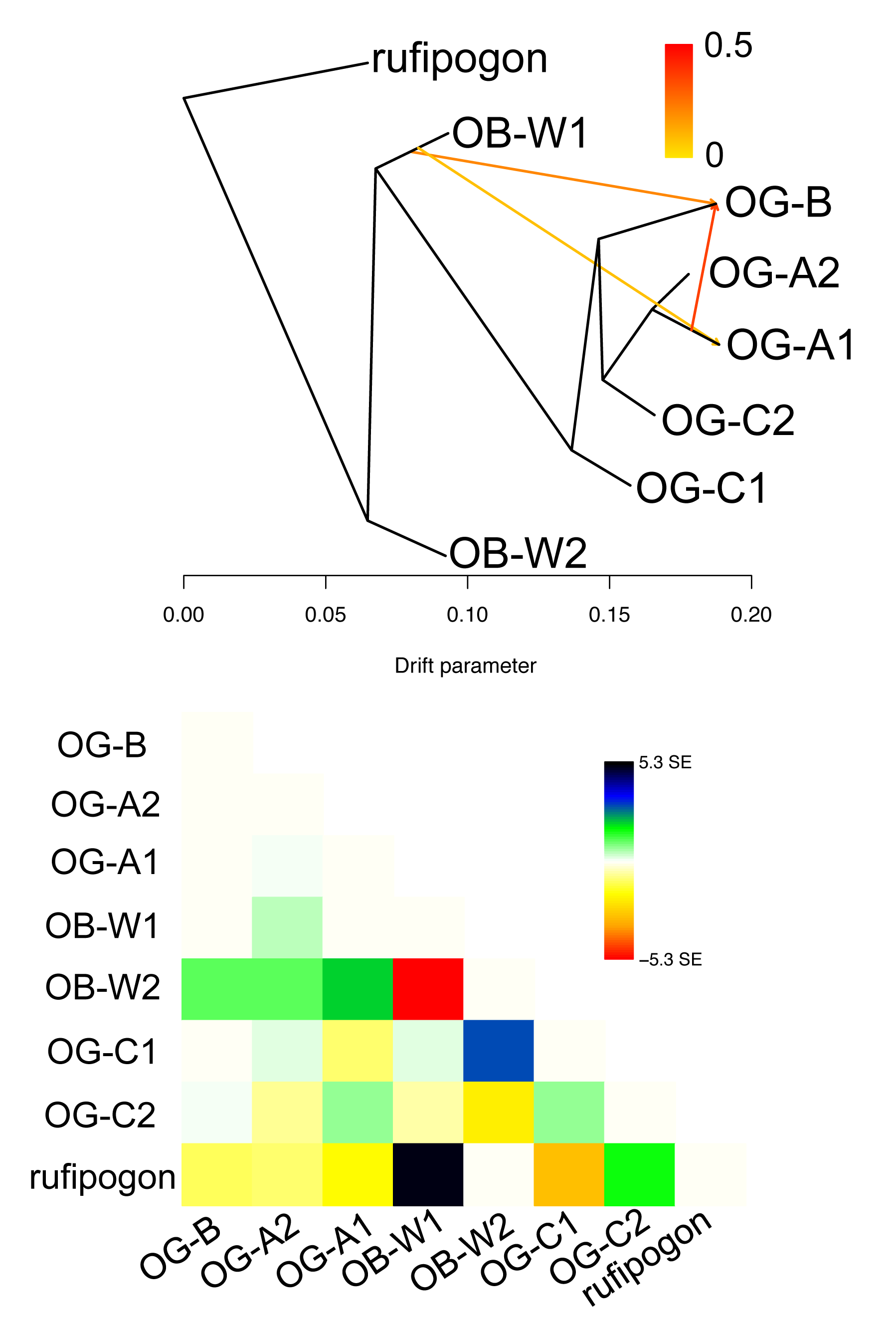

Supplement: S7 Fig — (TIF) [file pgen.1007414.s007.tif]

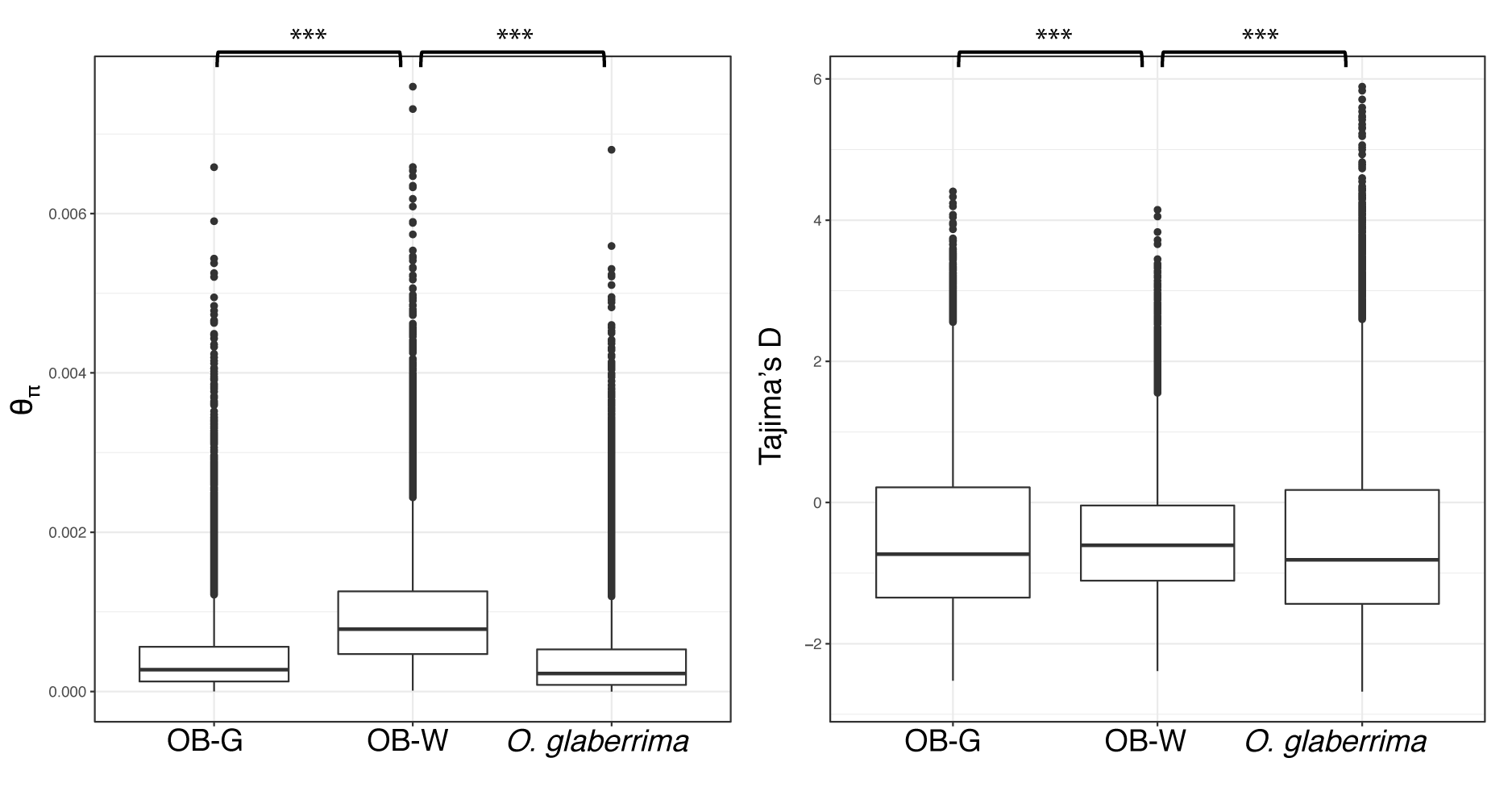

Supplement: S8 Fig — Significant difference after Mann-Whitney U test (p < 0.001) are indicated with three stars. (TIF) [file pgen.1007414.s008.tif]

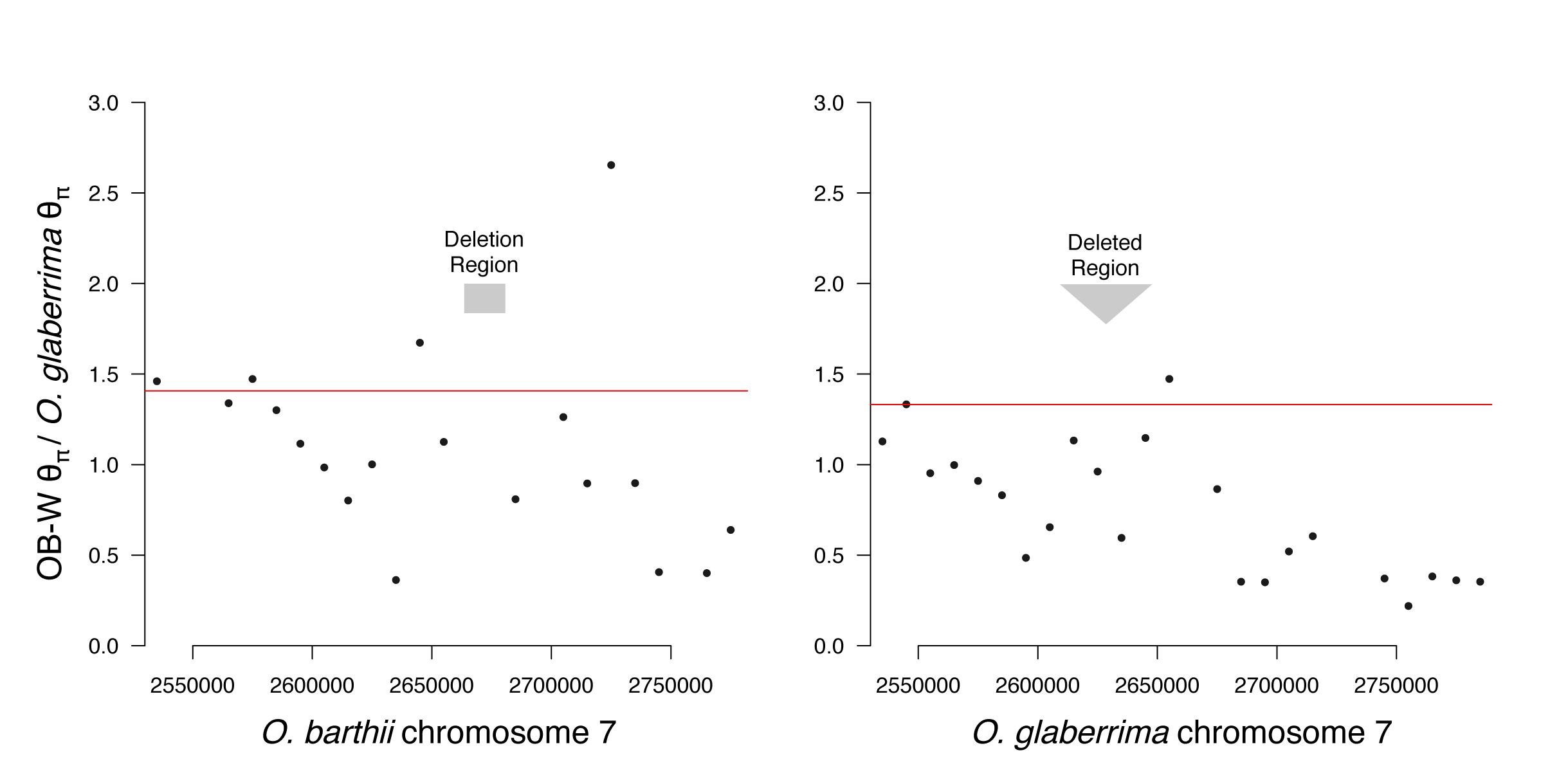

Supplement: S9 Fig — (TIF) [file pgen.1007414.s009.tif]

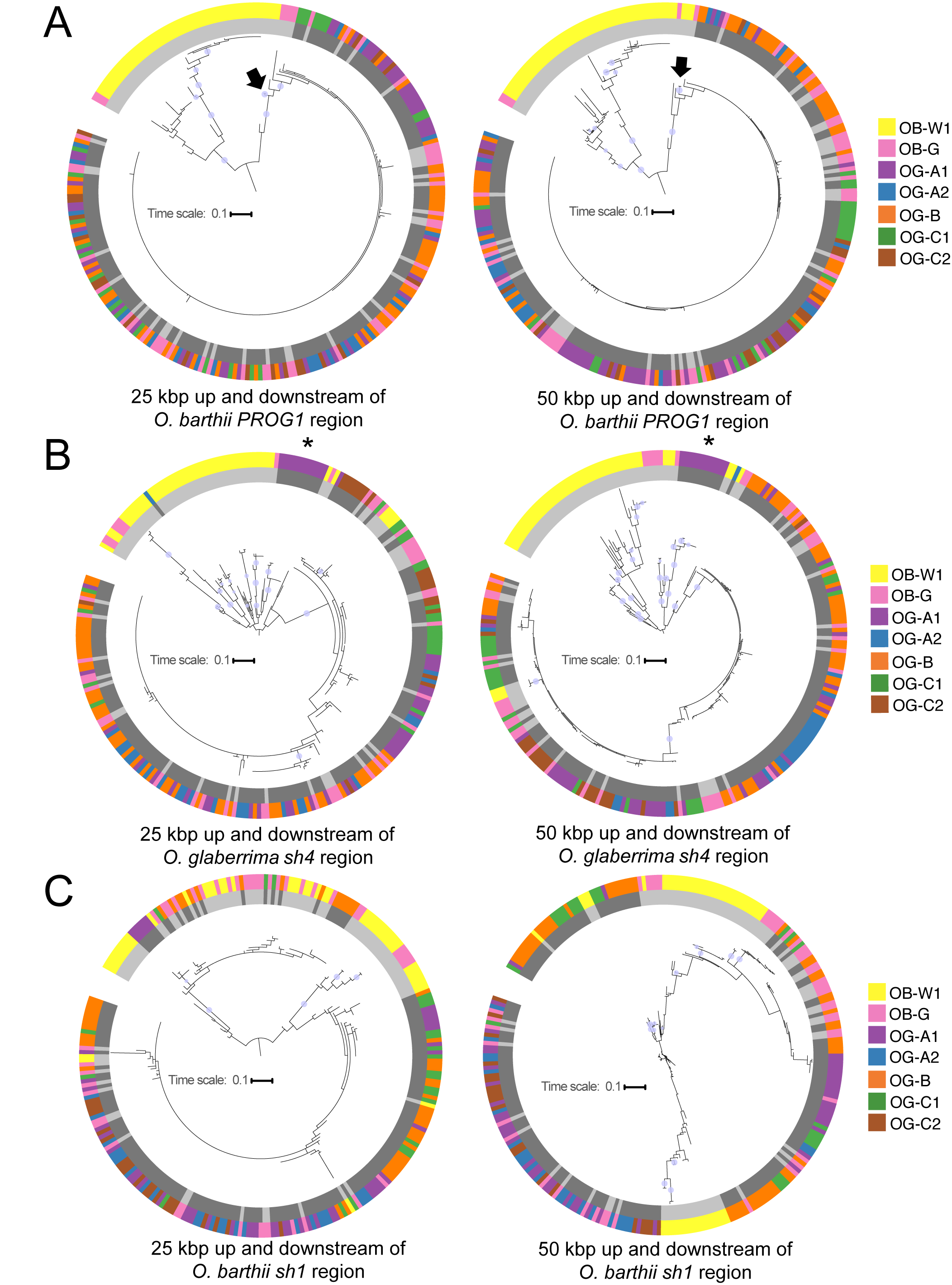

Supplement: S10 Fig — Maximum-likelihood tree of up and downstream 25 kbp or 50 kbp of the 3 domestication genes. Light grey represent O. barthii while dark grey represent O. glaberrima individuals. (A) Tree for PROG1 region. Black arrows indicate the two wild rice that are sister to all O. glaberrima samples. (B) Tree for sh4 region. Star indicates the individuals without the nonsense mutation. (C) Tree for sh1 region. Nodes with greater then 90% bootstrap support are shown with circles. (TIF) [file pgen.1007414.s010.tif]

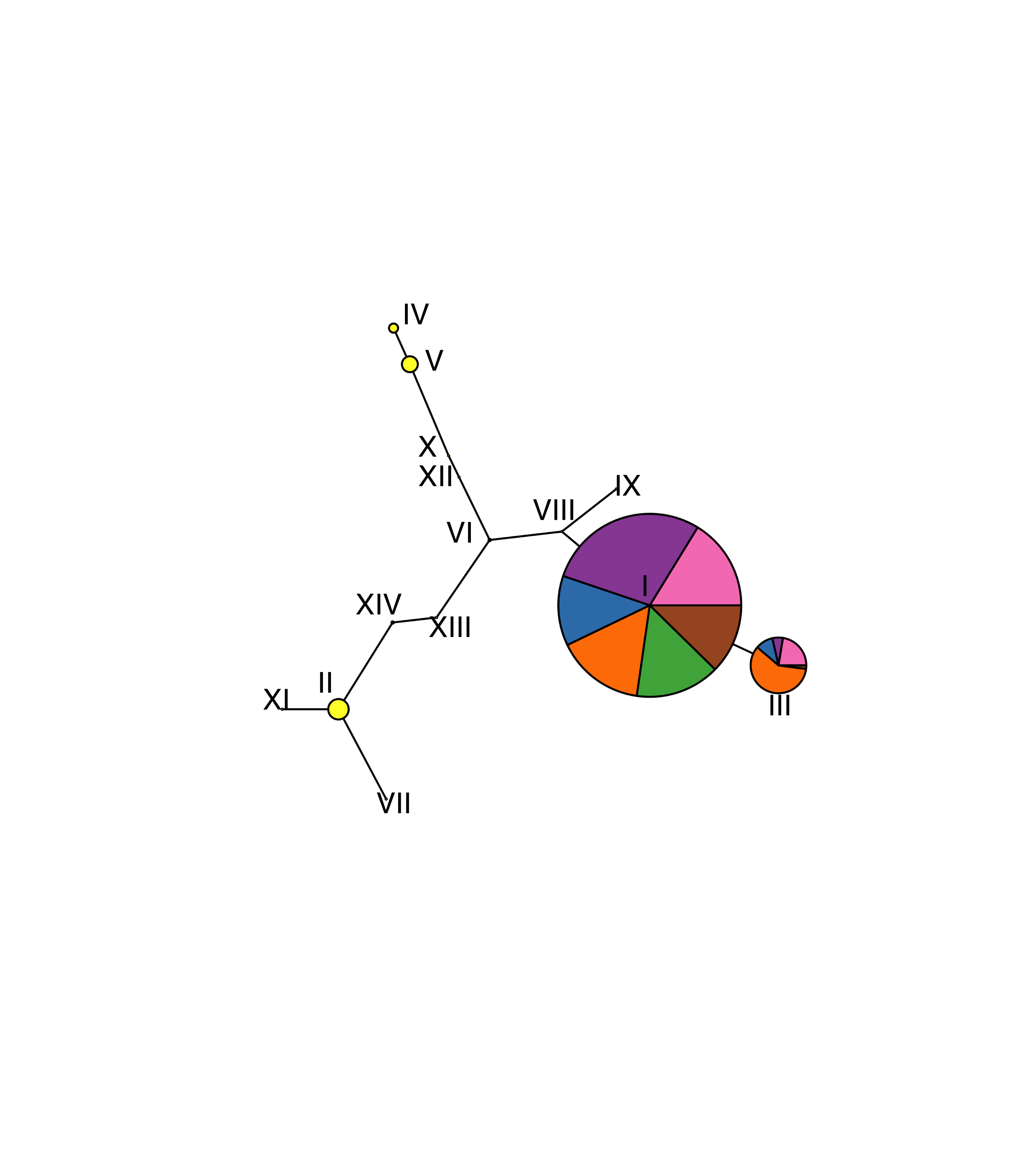

Supplement: S11 Fig — Haplotype network of the downstream 5 kbp of the PROG1 deletion. (TIF) [file pgen.1007414.s011.tif]

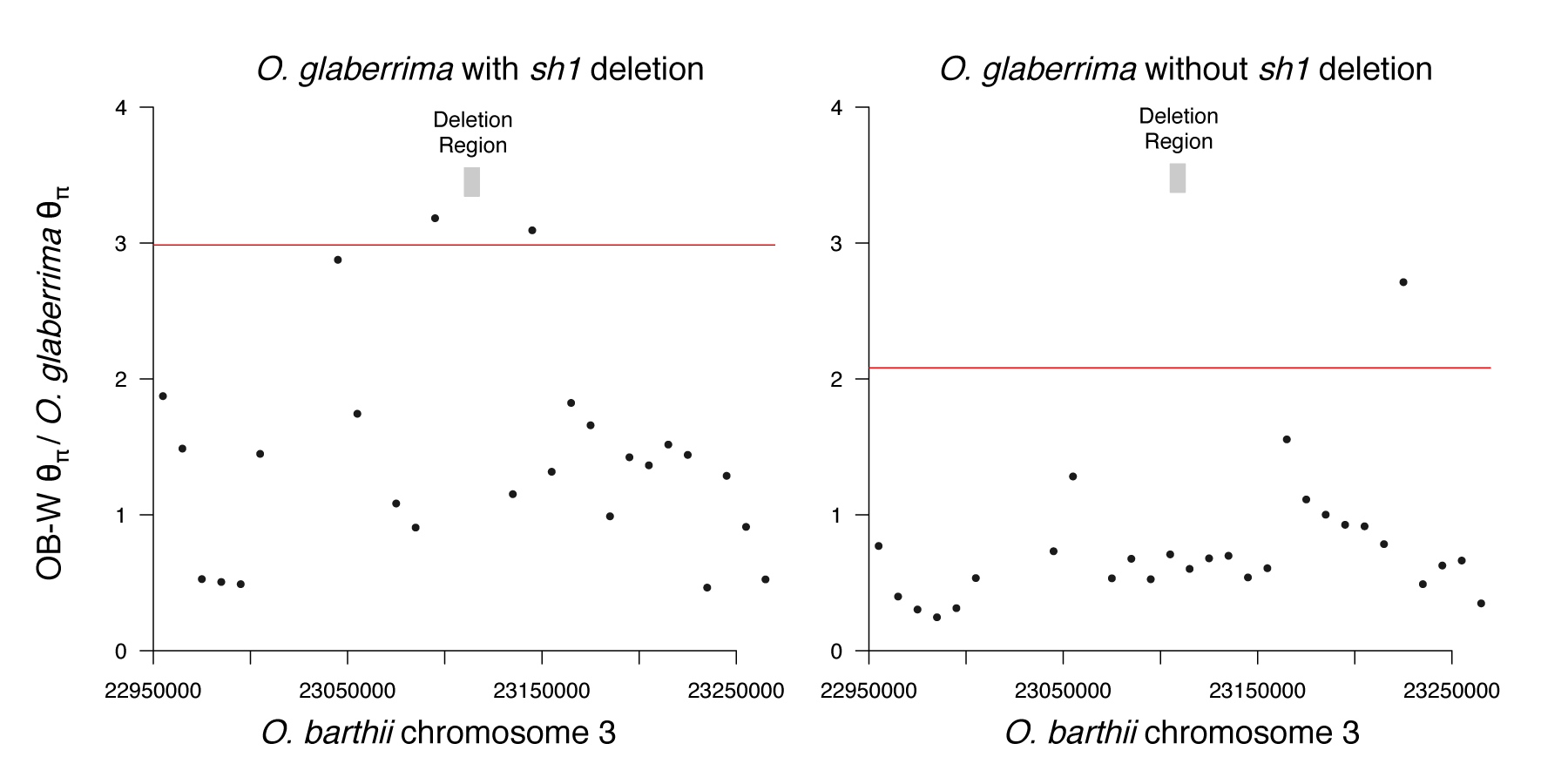

Supplement: S12 Fig — πw/ πD statistics around the sh1 region in O. barthii reference genome for O. glaberrima individuals with (left) and without (right) the sh1 deletion. (TIF) [file pgen.1007414.s012.tif]

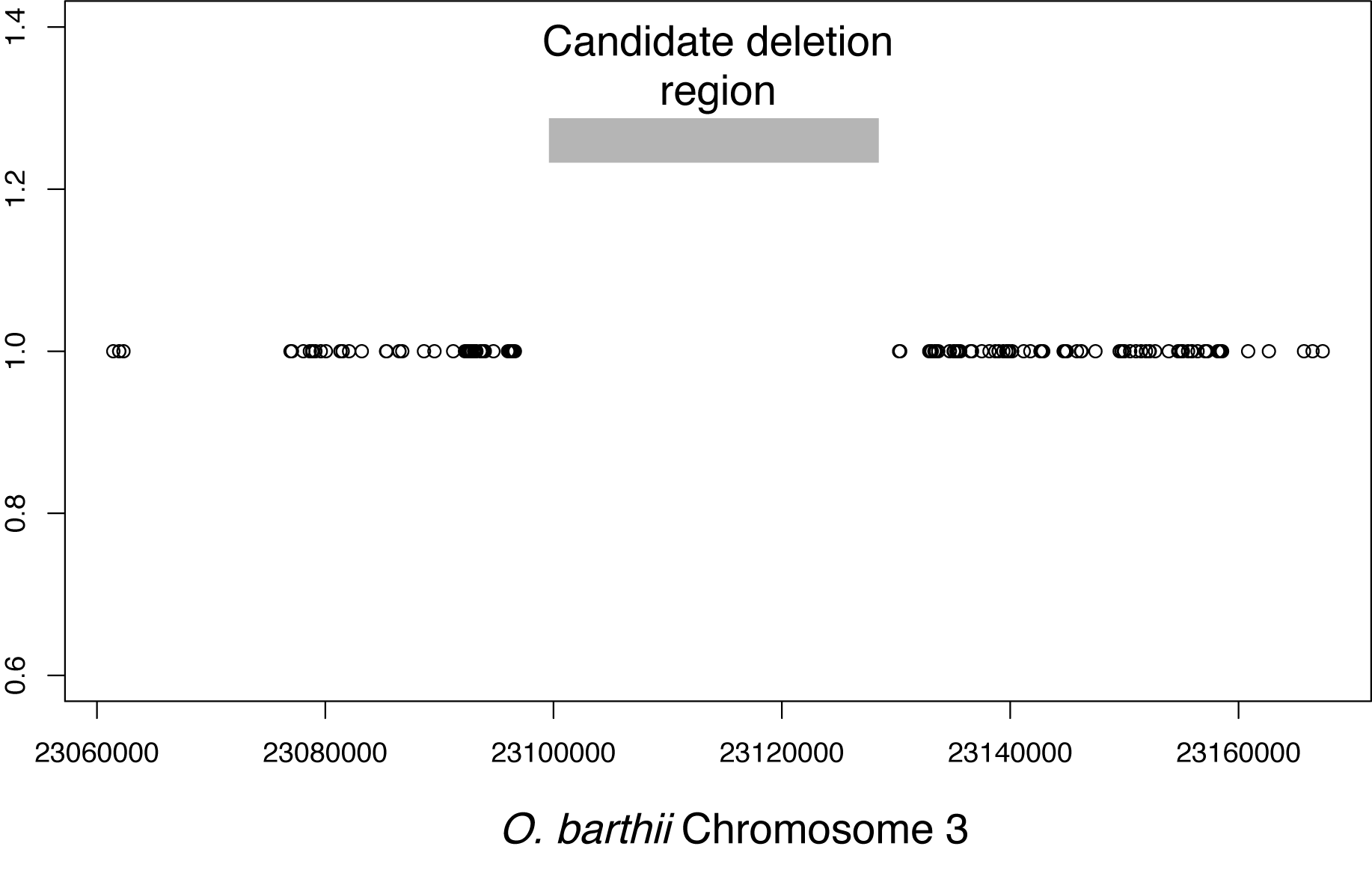

Supplement: S13 Fig — (TIF) [file pgen.1007414.s013.tif]

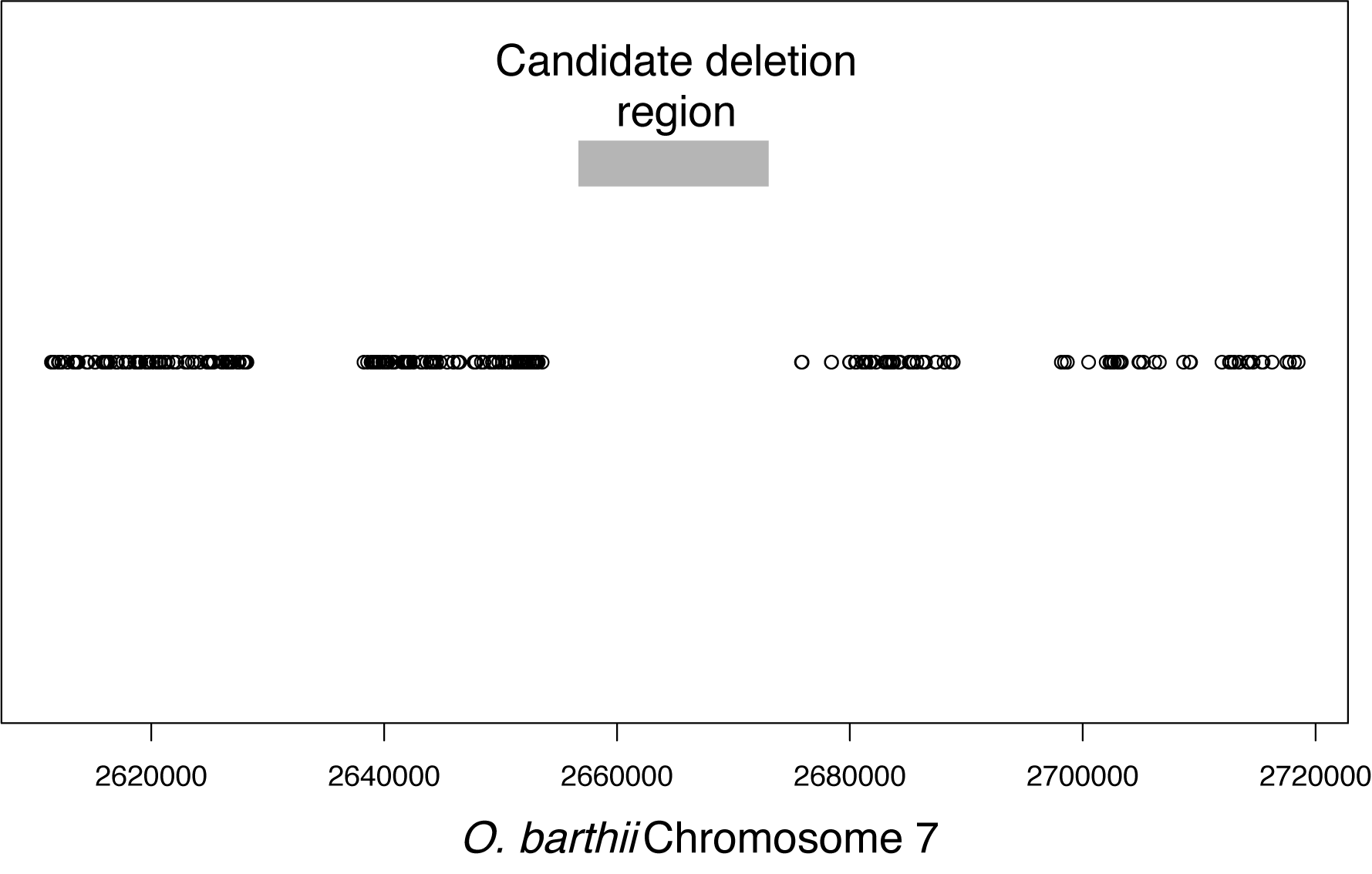

Supplement: S14 Fig — (TIF) [file pgen.1007414.s014.tif]

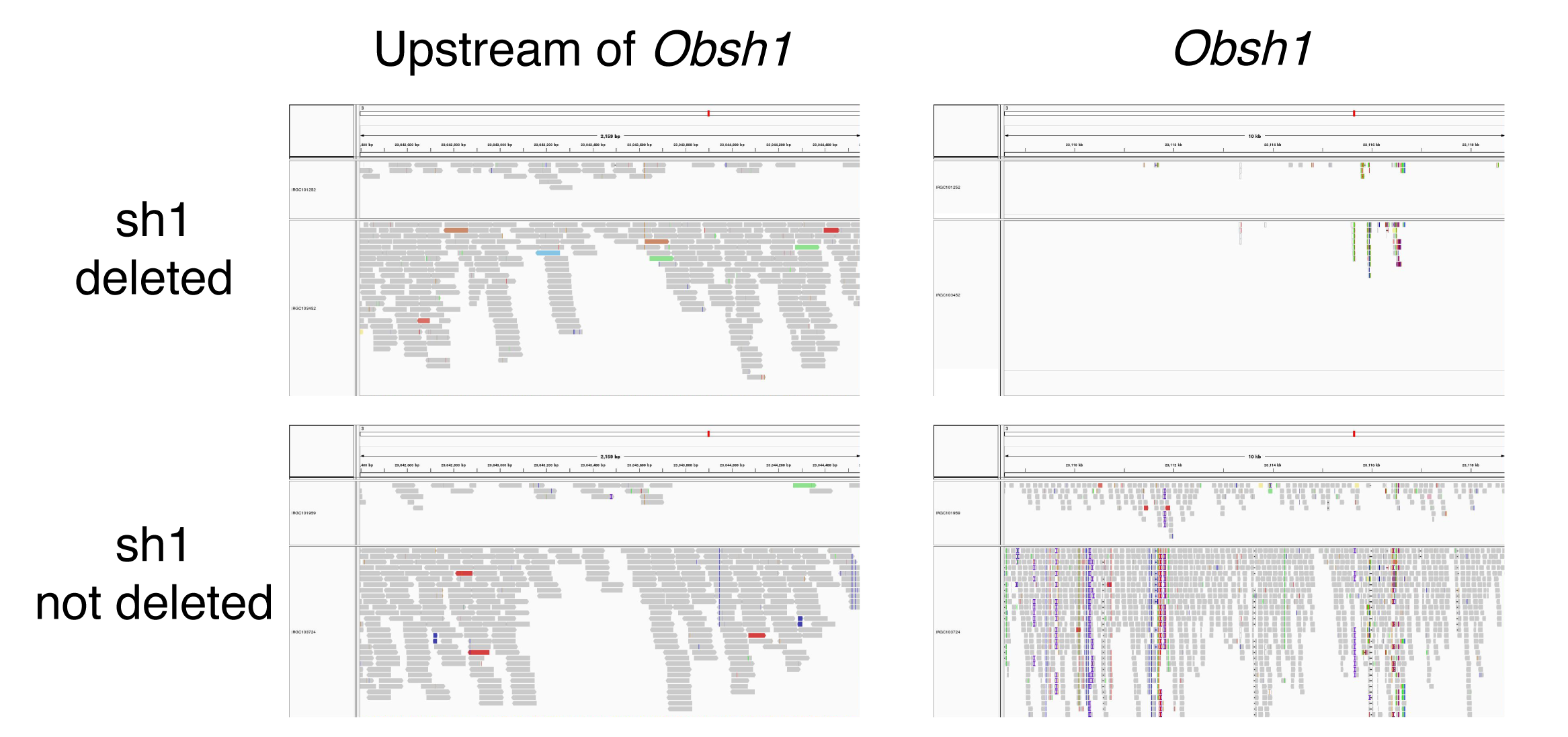

Supplement: S15 Fig — In each panel an individual with low and high coverage are compared. (TIF) [file pgen.1007414.s015.tif]
